# Chromatin lncRNA *Platr10* controls stem cell pluripotency by coordinating an intrachromosomal regulatory network

**DOI:** 10.1186/s13059-021-02444-6

**Published:** 2021-08-19

**Authors:** Zhonghua Du, Xue Wen, Yichen Wang, Lin Jia, Shilin Zhang, Yudi Liu, Lei Zhou, Hui Li, Wang Yang, Cong Wang, Jingcheng Chen, Yajing Hao, Huiling Chen, Dan Li, Naifei Chen, Ilkay Celik, Yanbo Zhu, Zi Yan, Changhao Fu, Shanshan Liu, Benzheng Jiao, Zhuo Wang, Hui Zhang, Günhan Gülsoy, Jianjun Luo, Baoming Qin, Sujun Gao, Philipp Kapranov, Miguel A. Esteban, Songling Zhang, Wei Li, Ferhat Ay, Runsheng Chen, Andrew R. Hoffman, Jiuwei Cui, Ji-Fan Hu

**Affiliations:** 1 Key Laboratory of Organ Regeneration and Transplantation of Ministry of Education, Stem Cell and Cancer Center, First Hospital, Jilin University, Changchun, Jilin 130061 People’s Republic of China; 2 Stanford University Medical School, VA Palo Alto Health Care System, Palo Alto, CA 94304 USA; 3 CAS Key Laboratory of RNA Biology, Institute of Biophysics, Chinese Academy of Sciences, Beijing, 100101 People’s Republic of China; 4 Department of Endocrinology, Xiangya Hospital, Central South University, Changsha, Hunan People’s Republic of China; 5 Google Inc., Mountain View, CA 94043 USA; 6 Institute of Genomics, School of Biomedical Sciences, Huaqiao University, Xiamen, 361021 People’s Republic of China; 7 Guangzhou Institutes of Biomedicine and Health, Chinese Academy of Sciences, Guangzhou, Guangdong 510530 People’s Republic of China; 8 La Jolla Institute for Allergy and Immunology, La Jolla, California 92037 USA

**Keywords:** Stem cell, Pluripotency, Long noncoding RNA, Intrachromosomal loop, *Oct4*, *Sox2*, DNA methylation

## Abstract

**Background:**

A specific 3-dimensional intrachromosomal architecture of core stem cell factor genes is required to reprogram a somatic cell into pluripotency. As little is known about the epigenetic readers that orchestrate this architectural remodeling, we used a novel chromatin RNA in situ reverse transcription sequencing (CRIST-seq) approach to profile long noncoding RNAs (lncRNAs) in the *Oct4* promoter.

**Results:**

We identify *Platr10* as an *Oct4* - *Sox2* binding lncRNA that is activated in somatic cell reprogramming. *Platr10* is essential for the maintenance of pluripotency, and lack of this lncRNA causes stem cells to exit from pluripotency. In fibroblasts, ectopically expressed *Platr10* functions in trans to activate core stem cell factor genes and enhance pluripotent reprogramming. Using RNA reverse transcription-associated trap sequencing (RAT-seq), we show that *Platr10* interacts with multiple pluripotency-associated genes, including *Oct4*, *Sox2*, *Klf4*, and *c-Myc*, which have been extensively used to reprogram somatic cells. Mechanistically, we demonstrate that *Platr10* helps orchestrate intrachromosomal promoter-enhancer looping and recruits TET1, the enzyme that actively induces DNA demethylation for the initiation of pluripotency. We further show that *Platr10* contains an *Oct4* binding element that interacts with the *Oct4* promoter and a TET1-binding element that recruits TET1. Mutation of either of these two elements abolishes *Platr10* activity.

**Conclusion:**

These data suggest that *Platr10* functions as a novel chromatin RNA molecule to control pluripotency *in trans* by modulating chromatin architecture and regulating DNA methylation in the core stem cell factor network.

**Supplementary Information:**

The online version contains supplementary material available at 10.1186/s13059-021-02444-6.

## Introduction

Terminally differentiated cells can be reprogrammed into a pluripotent stage known as induced pluripotent stem cells (iPSCs) by using a cocktail of stem cell transcription factors *Oct4-Sox2-Klf4-c-Myc* (OSKM) [1], small chemical compounds [2, 3], or by nuclear transfer [4]. However, these reprogramming processes are extremely inefficient and time-consuming, hindering potential clinical applications of iPSCs for regenerative medicine [5].

It is now clear that there are strong epigenetic barriers that must be overcome before cells achieve full pluripotency. The initiation of cell reprogramming towards pluripotency requires appropriate expression of the core stem cell factor network [6, 7]. The specific chromatin architecture surrounding key pluripotency gene loci, such as *Oct4*, *Sox2*, and *Nanog*, is the culmination of critical epigenetic steps involved in the regulation of cell remodeling [8]. Self-renewal of pluripotent stem cells also requires the formation of a specific long-range interchromosomal and intrachromosomal interacting network.

To explore the mechanisms underlying reprogramming, we compared promoter DNA binding and chromatin architecture between iPSCs that have completed reprogramming and cells we referred to as “non-iPSCs,” which expressed lentiviral OSKM factors, but failed to complete reprogramming [9]. We found that the virally expressed OSKM factors bound to their target genes in both groups of cells. However, in non-iPSCs, the target genes could not be activated to achieve pluripotency, partially due to the lack of a promoter-enhancer intrachromosomal loop architecture [9]. Formation of this intrachromosomal loop is a critical epigenetic barrier that must be overcome for the induction of pluripotency. In addition, the maintenance of pluripotency also requires a specific, higher-order genomic architecture consisting of long-range chromatin interactions [10]. However, the molecular factors that orchestrate this pluripotency-specific intrachromosomal network are still uncharacterized.

Recent studies suggest that long noncoding RNAs (lncRNAs) are important structural components of three-dimensional nuclear architecture [11, 12]. In the nucleus, lncRNAs can regulate gene transcription at different functional steps *in cis* or *in trans* through multiple epigenetic mechanisms, including binding to regulatory elements (promoters and enhancers), inter-and intrachromosomal interactions, histone modifications, DNA methylation, chromatin remodeling [13], and post-transcriptional protein ubiquitination and degradation [14]. Recently, we used a chromatin RNA in situ reverse transcription sequencing (CRIST-seq) approach to map the lncRNA network within regulatory elements of key stemness genes [15]. In the present study, we have employed this approach to discover which lncRNAs interact with the *Oct4* promoter. Activation of this master stem cell factor is absolutely required for the establishment and maintenance of pluripotency [9]. We hypothesized that lncRNAs embedded in this chromatin structure might actively participate in the control of the *Oct4* promoter.

Using this approach, we have identified 27 differentially transcribed RNA candidates that interacted with the *Oct4* and *Sox2* promoters [15]. Among them, *Platr10* is a lncRNA that is co-expressed with *Oct4* and other pluripotency factors [16]. In this report, we focus on the mechanisms underlying the role of *Platr10* in reprograming. We demonstrate that *Platr10* is primarily located in the nucleus, where it regulates pluripotency by binding to multiple stem cell core factors, including *Oct4*, *Sox2*, *Klf4*, and *c-Myc*, four factors that have been used to induce pluripotent reprogramming. Mutation assays demonstrate that *Platr10* contains an *Oct4* binding element as well as a TET1 binding element, both of which are required for the regulation of stem cell pluripotency and reprogramming.

## Results

### Profiling pluripotency-associated lncRNAs by CRIST-seq

**Fig. 1:**
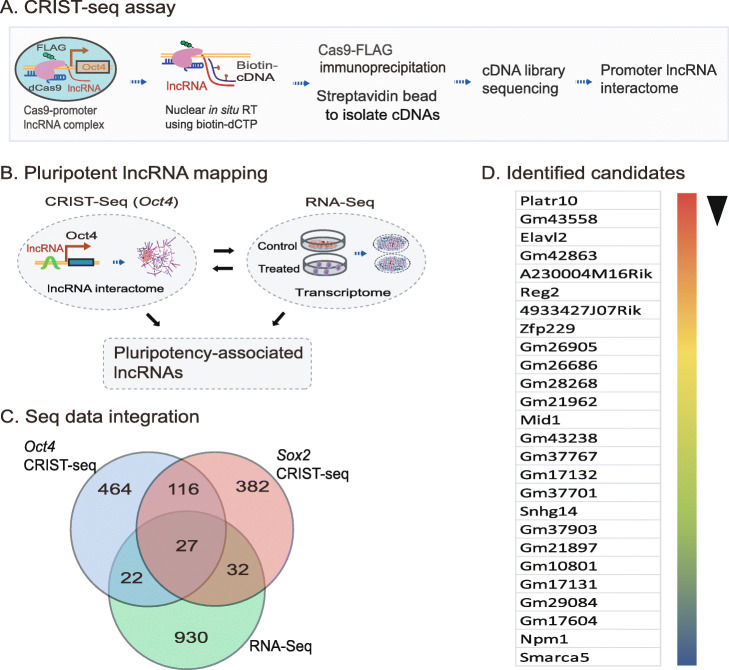
Profiling of *Oct4* promoter-interacting lncRNAs by CRIST-seq. **A** Schematic diagram of the chromatin-lncRNA in situ reverse transcription trap sequencing (CRIST-Seq) assay. dCas9: Catalytically inactive CRISPR Cas9; FLAG: a tag octapeptide having the sequence motif DYKDDDDK that is attached to the N-terminal of Cas9; *Oct4*-gRNA: Cas9 guiding RNAs that target the *Oct4* promoter. In iPSCs, Cas9-gRNA binds to the *Oct4* promoter through a mechanism of base pairing between the gRNA and target DNA. After fixation, the *Oct4* promoter-interacting RNAs were reverse transcribed into cDNAs in the isolated nuclei with biotin-dCTP. The Cas9 *Oct4* promoter biotin-cDNA complex was immunoprecipitated by a Cas9-FLAG antibody, and biotin-cDNAs were further purified from genomic DNAs by biotin-streptavidin bead purification. The CRIST-captured cDNAs were profiled by Illumina library sequencing to identify the RNA components that regulate pluripotency. **B** Profiling pluripotency-associated lncRNAs by the combined CRIST-seq and RNA-Seq datasets. The *Oct4*-interacting lncRNAs identified by CRIST-seq were integrated with the dataset of RNA transcriptome sequencing. The combination of these two datasets identifies lncRNAs that not only interact with the *Oct4* promoter but are also expressed differentially in reprogramming. **C** Integration of the RNA-Seq and CRIST-Seq datasets. RNA-Seq was initially used to identify the upregulated RNAs (>2-fold, *p*<0.05) in reprogramming. The upregulated RNAs were then integrated into the *Oct4* and *Sox2* CRIST-Seq datasets using a VENN program. The CRIST-Seq data were adjusted over the values of the IgG control and Cas9-gCT control. A cut-off threshold of peak enrichment FPKM>50 was arbitrarily set to select CRIST-Seq RNAs for VENN analysis. Integration of three datasets generated a list of 27 pluripotency-associated RNA candidates. **D** A list of 27 pluripotency-associated RNA (PALR) candidates identified by RNA-Seq and CRIST-Seq. The RNA candidates are ranked based on the RNA expression-fold from high (red) to low (blue) between fibroblasts (FBC) and iPSCs

To identify epigenetic pathways that coordinate chromatin remodeling, we focused on lncRNAs that interact with the *Oct4* promoter, a core stem cell factor that is essential for pluripotency maintenance. We hypothesized that components that interact with the *Oct4* promoter, particularly those lncRNAs that are specifically transcribed during reprogramming, would participate in the regulation of pluripotency. We used a CRIST-Seq approach [15] to profile lncRNAs that interact with the *Oct4* promoter (Fig. 1A). This assay combines the simplicity of nuclear in situ RNA biotin labeling with the specificity of CRISPR Cas9 gene targeting. The assay includes (1) targeting of the promoter complex by Cas9 gRNAs, (2) RNA in situ labeling by reverse transcription with biotin-dCTP, (3) pull-down of the locus and its associated cDNAs by Cas9-FLAG immunoprecipitation, (4) purification of the promoter-associated cDNAs from genomic DNAs by streptavidin beads, and (5) Illumina cDNA library sequencing (Fig. S1).

To profile the *Oct4*-associated lncRNAs, two Cas9 gRNAs were designed from the *Oct4* promoter (Fig. S2) and cloned into a Cas9-FLAG lentiviral vector (Fig. S1B). After lentivirus infection and puromycin selection, iPSCs were cross-linked with formaldehyde to fix the *Oct4* promoter-RNA chromatin structure. The chromatin-associated RNAs were then in situ reverse transcribed in the nucleus into cDNAs using biotin-dCTP. The Cas9-FLAG-Oct4-cDNA complex was immunoprecipitated by an anti-FLAG antibody. After reversal of the crosslinks, the biotin-labeled cDNAs were purified with streptavidin beads and used for Illumina library sequencing to identify the *Oct4*-interacting RNA network [15].

We used quantitative PCR to examine the specificity of CRIST targeting (Figs. S1C-S1E). We detected the enrichment of CRIST signals at the targeting site (pOct4), where the two gRNAs are located. At the same locus, no enrichment was detected for a random gRNA control (gCT) or a Cas9 vector control (Vector). Similarly, we did not detect CRIST enrichment at the 5′-control site (5′-Ct), which is 13.9 kb distant from the p*Oct4* target site. In addition, no Cas9-gRNA enrichment was detected at the off-target control site that is 33.8 kb upstream of the housekeeping gene GAPDH. Taken together, these data demonstrate the specificity of the CRIST approach to target the *Oct4* promoter.

We then mapped the RNA network in the *Oct4* promoter. Two controls were performed in parallel with *Oct4* CRIST sequencing, including a random Cas9 gRNA control (gCT) and an IgG immunoprecipitation control (IgG). To define the specific binding of RNAs, CRIST-Seq signal intensities were normalized over that of the non-targeting Cas9 gCT control and the IgG control using parameters of fold change ≥2 and *p* value < 0.05.

LncRNAs are critical components of the regulatory chromatin complex. Like chromatin factors, some lncRNAs may regulate multiple gene targets. Thus, we proposed to identify those lncRNAs that bind to multiple pluripotent target genes, assuming that they would play more important roles in pluripotency than those that bind only to a single gene target. Thus, we integrated the above *Oct4* CRIST-seq data with the CRIST-seq data targeting *Sox2* [15], a second core stem cell transcription factor that is essential for the maintenance of pluripotency (Fig. S3).

We reasoned that a key lncRNA candidate should also become activated during reprogramming. Therefore, we collected cells at different stages of reprogramming [9, 17]. RNA-Seq was performed to identify RNAs that are differentially expressed in association with reprogramming. RNA transcriptome sequencing identified differentially expressed lncRNAs (>2 fold) between control fibroblasts and iPSCs [18]. To identify the pluripotency-associated lncRNA candidates, we integrated the *Oct4* and *Sox2* CRIST lncRNA data with the RNA-Seq data (Fig. 1B, C). By combining these datasets, we identified 27 RNA candidates that not only interacted with the *Oct4* and *Sox* promoters, but are also differentially transcribed during reprogramming into pluripotency (Fig. 1D) [15].

### CRIST-seq identifies Platr10 as an essential lncRNA for pluripotency

The lncRNA NONMMUT043505 (*Platr10*) showed the largest fold increment when fibroblasts were reprogrammed into iPSCs. This lncRNA is located on chromosome 3, and RNA-seq data showed that it is specifically expressed in iPSCs (Fig. S4). Further analysis showed that this lncRNA was comprised of two variants (Fig. S5). Neither variant contains a large open reading frame. Variant 1, which contains 4 exons, is the major transcript, while variant 2 contains three exons; the latter variant matches with *Platr10*, one of 32 pluripotency-associated lncRNAs (*Platrs*) identified by Bergmann et al. [16]. Using a weighted co-expression analysis, they showed that *Platrs* were clustered tightly with the expression *Oct4* and other pluripotency factors. We thus focused on *Platr10* and examined its underlying mechanisms in pluripotency.

**Fig. 2:**
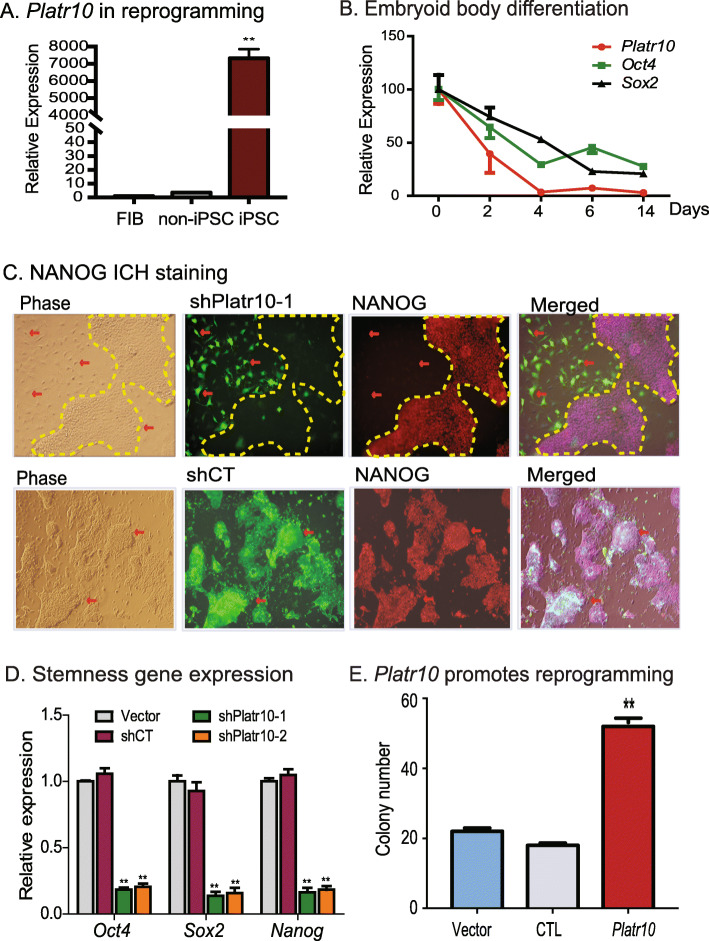
*Platr10* is required for the maintenance of pluripotency. **A** Reactivation of *Platr10* in reprogramming. Skin fibroblasts were reprogrammed using lentiviral *Oct4*-*Sox2*-*Kilf4-c-Myc* (OSKM). Cells were collected at different stages of reprogramming and the expression of *Platr10* was measured by RT-PCR. FIB, fibroblasts; iPSC, induced pluripotent stem cells; non-iPCS (un-reprogrammed cells), cells that express the four viral OSKM factors, but fail to complete reprogramming. β-Actin was used as the PCR control. Throughout the manuscript, the data are presented as the mean ± SD from three independent experiments unless they are specifically defined. **p < 0.01 as compared with FIB and non-iPSCs. **B** *Platr10* expression is associated with *Sox2* and *Oct4* expression during embryoid body (EB) differentiation. iPSCs were collected at different stages of EB formation for quantitative PCR. **C** Requirement for *Platr10* in the maintenance of pluripotency. shPlatr10-1, shRNA vector that targets *Platr10* lncRNA; shCT, random shRNA control; Vector, lentiviral vector control. *Platr10* was knocked down by shRNA lentiviruses in E14 cells. Cells transfected with lentiviruses carrying a random shRNA (gCT) were used as the control. The lentivirus-transfected cells were tracked by the co-expressed copGFP. Pluripotency status was examined by immunohistochemical (IHC) staining of stem cell marker NANOG. Note that the exit of iPSCs from pluripotency in the shRNA-copGFP expressing cells is accompanied by altered cell morphology and the loss of NANOG protein (red arrow). The cell islands that escape lentiviral shPlatr10 transfection are marked by a yellow dotted line. These cells maintain the same stem cell pluripotency as the iPSCs. **C** *Platr10* is essential for optimal activity of core stem cell factor genes in iPSCs. After lentiviral transfection, iPSCs were selected by puromycin. The mixed stable cells were collected for qPCR quantitation. **p < 0.01 as compared with the Vector and shCT controls. **D** *Platr10* enhances cell reprogramming. MEF cells were transfected with the lentiviruses carrying *Platr10*, the empty vector (Vector), and CTL (lncRNA control containing *Platr10* antisense RNA). After doxycycline (DOX) induction, iPSC colonies were detected using an alkaline phosphatase (AP) staining kit and were quantitated as iPSC colonies per microscope field. **p < 0.01 as compared with the Vector and CTL controls

We first quantitated the transcriptional abundance of *Platr10* in cells collected at different stages of reprogramming, including fibroblast control, fully reprogrammed cells (iPSCs), and “non-iPSCs” that expressed the lentiviral OSKM factors but failed to complete reprogramming. *Platr10* expression correlated with pluripotency status, as it was silenced in fibroblasts and transcribed at only a very low level in non-iPSCs. The expression of *Platr10* was greatly increased in cells that were fully reprogrammed (iPSCs) (Fig. 2A). Using sodium bisulfite sequencing, we found that the *Platr10* gene was epigenetically regulated by DNA methylation (Fig. S6). The CpG islands in the *Platr10* promoter were hypermethylated in fibroblasts but unmethylated in iPSCs, suggesting an epigenetic regulation of this lncRNA in reprogramming.

We also collected cells during embryoid body differentiation. Using quantitative PCR, we found that *Platr10* became significantly downregulated during embryoid body differentiation, in parallel with core stem cell factors *Oct4*, *Sox2*, and *Nanog* (Fig. 2B). We then examined the function of *Platr10* by transfecting E14 cells with two shRNA lentiviruses (Fig. S7A, shPlatr10-1 and shPlatr10-2). Both shRNA lentiviruses significantly knocked down *Platr10* lncRNA as compared with the random control (shCT) and vector control (Fig. S7B). We examined if *Platr10* knockdown would affect cell morphology of iPSCs. The activity of the CMV promoter-copGFP was much weaker in iPSCs than it was in differentiated cells. In the random shRNA control group (shCT), the copGFP-positive cells maintained the same cell morphology as pluripotent stem cells (Fig. 2C, bottom panels). However, knockdown of *Platr10* dramatically altered cell morphology (top panels, red arrows, shPlatr10-1). The *Platr10*-deficient cells became enlarged and flat, appearing more like fibroblasts.

We then examined the pluripotency of treated iPSCs by immunohistochemical staining of pluripotency-associated marker protein NANOG. The shCT control cells showed extensive expression of the pluripotency-associated marker protein NANOG (Fig. 2C, bottom panel 3). After *Platr10* shRNA knockdown, iPSCs became differentiated and lost the pluripotency-associated marker NANOG (top panel; unmarked regions, red arrow). Interestingly, in the shPlatr10 group, there are some “island” cells that escaped lentiviral infection and did not express the shPlatr10-copGFP track marker. These cells served as the “escaped” control and still maintained the original compact shape of iPSCs and expressed NANOG (yellow marked areas without copGFP fluorescence).

Using qPCR, we found that knockdown of *Platr10* was associated with downregulation of the three core stem cell factor genes *Oct4*, *Sox2*, and *Nanog*. In the control groups, treatment with a random control shRNA (shCT) and the vector control (Vector) did not affect the activity of core stem cell factor genes (Fig. 2D).

We further examined the role of *Platr10* in pluripotent reprogramming using a DOX-inducible system [19]. OG2 MEF cells were first transfected with lentiviruses carrying the *Platr10* cDNA, DsRed control (CTL), and empty vector (Vector). After puromycin selection, cells were incubated in reprogramming media containing the doxycycline (DOX) inducer and then stained for pluripotent marker NANOG. Compared with the vector (Vector) and DsRed (CTL) controls, ectopic expression of *Platr10* was associated with increased reprogramming of MEF cells into pluripotency as quantitated by alkaline phosphatase (AP) staining (Fig. 2E) and NANOG foci (Fig. S7C).

We also validated the role of *Platr10* using CRISPR Cas9 editing. For this, we constructed two targeting vectors carrying the dual SpCas9-NmCas9 cassette [20] and four gRNAs to target the *Platr10* promoter and 3′-downstream region, respectively (Fig. S8A). The donor vector was constructed to include two *Platr10* arms for homologous recombination, copGFP/Puro for the positive selection, and TK for the negative selection. Using this dual Cas9 approach, we successfully deleted the region covering the *Platr10* promoter and coding region in E14 cells. The homozygous deletion of *Platr10* was confirmed by DNA sequencing. As expected, *Platr10*-deleted cells (GFP-positive) showed a change in morphology (Fig. S8B) and exhibited loss of pluripotent marker NANOG immunostaining (Fig. S8C). As the control, the random Cas9 gRNAs did not alter the immunostaining signal of NANOG in E14 cells. Taken together, these data suggest that *Platr10* is critical for the maintenance of pluripotency.

### Platr10 binds to the Oct4 promoter using a 50 bp OBE element

We then explored the mechanisms underlying the role of *Platr10* in pluripotency. The CRIST-seq IGV analysis also revealed that *Platr10* bound to the *Oct4* promoter using a consensus 50 bp fragment located in exon 3 (5′-GACAAAAAATGGAGCAGACTGAAGGAAAGGCCATCCAGAGACTGTCCCAC-3′) (Fig. S9). Using a cellular fractionation assay, we showed that *Platr10* was primarily localized to the nucleus (Figs. S10A-10B). The nuclear *Platr10* was primarily in the chromatin-bound form (Fig. S10C). An RNA fluorescent in situ hybridization (FISH) assay also validated the nuclear localization of *Platr10* lncRNA (Fig. S10D).

### Platr10 binds to multiple stem cell core factor genes

**Fig. 3:**
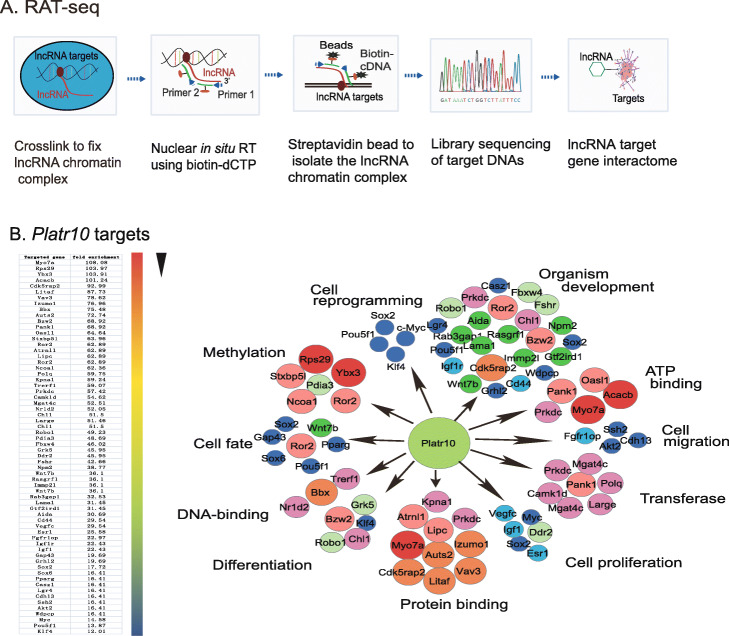
Genome wide mapping of the *Platr10* target gene network by RAT-seq. **A** Schematic diagram of the RNA reverse transcription-associated trap sequencing (RAT-seq) assay. *Platr10* lncRNA was labeled with biotin-dCTP in situ reverse transcription using three *Platr10*-specific complementary primers. The biotin-*Platr10* chromatin complex was isolated by streptavidin beads and the *Platr10*-binding target DNAs were isolated for Illumina library sequencing. This RAT-seq generates a *Platr10* genome wide target DNA network. **B** *Platr10* target gene interacting network. The *Platr10* target pathway genes were mapped by gene ontology analysis. *Platr10* binds to all four stem cell-associated factors that have been used to reprogram somatic cells: *Oct4*, *Sox2*, *Klf4*, and *c-Myc*

We hypothesized that *Platr10* might control reprogramming by regulating a target gene network that is associated with pluripotency. To test this hypothesis, we utilized RNA reverse transcription-associated trap sequencing (RAT-seq) [21, 22] to identify a gene network coordinated by *Platr10* (Figs. 3A). After crosslinking the chromatin structure, *Platr10* was reverse transcribed in situ under a more stringent condition using three *Platr10*-specific complementary primers (Table S1) and biotin-dCTP. The biotin-labeled *Platr10* chromatin complex was pulled down with streptavidin beads. Target gene DNAs were eluted, and a DNA library was constructed for Illumina sequencing. The *Platr10* RAT-seq data were adjusted over the random oligonucleotide RAT-seq data. Using parameters of fold change difference >2 and *p* value < 0.05, with false discovery rate (FDR) <0.1, we identified 416 *Platr10* target genes. Ontology analysis showed that *Platr10* bound to gene targets belonging to pathways that are closely related to stem cell maintenance and differentiation (Fig. 3B).

*Platr10* interacts with multiple core stem cell factor genes, including *Oct4*, *Sox2*, *Klf4*, and *c-Myc* (Fig. S11). These four transcription factors have been commonly used to reprogram somatic cells into pluripotency. These interactions were not detectable in a RAT-seq dataset from the RAT-seq control library (RAT-CT), in which two random oligonucleotides, instead of *Platr10*-specific antisense oligonucleotides, were used for the RAT assay. Neither did we detect the RAT-seq signal of stemness gene binding for the lncRNA control *Palr35*, which was also differentially expressed in reprogramming, with abundant transcripts in iPSCs. These data suggest that *Platr10* may actively and specifically participate in reprogramming by regulating pluripotency-associated transcriptional factor genes.

We further validated the *Platr10-Oct4* interaction in shPlatr10-treated E14 cells. Using RAT-qPCR, we detected a higher *Platr10-Oct4* interaction signal in control E14 cells (shCT) (Fig. S12A). After *Platr10* knockdown, however, only the background interaction signal was detected in shPlatr10-1 and shPlatr10-2 groups. Similarly, a very low background RAT signal was detected in the control lncRNA *Palr35*, which was also differentially expressed in reprogramming, but was not found in the list of CRIST-seq. We also validated this *Platr10-Oct4* promoter interaction using a ChIRP (Chromatin Isolation by RNA Purification) assay (Fig. S12B). This interaction was abolished in the shPlatr10 groups.

### Platr10 orchestrates pluripotency-specific intrachromosomal looping

Epigenetic remodeling plays a key role in cell-fate conversion. During chromatin remodeling, the genome undergoes major epigenetic alterations to reacquire the euchromatin characteristic of pluripotent cells. By comparing local chromatin structure of the *OCT4* locus, we previously showed that there is a pluripotency-associated intrachromosomal loop in iPSCs that juxtaposes a downstream enhancer to the gene’s promoter, enabling activation of endogenous stemness genes to achieve reprogramming [9]. Since *Platr10* binds to the same regulatory elements that are known to be involved in chromatin three-dimensional structure, we hypothesized that *Platr10* might be a critical factor that orchestrates intrachromosomal looping for reprogramming.

**Fig. 4:**
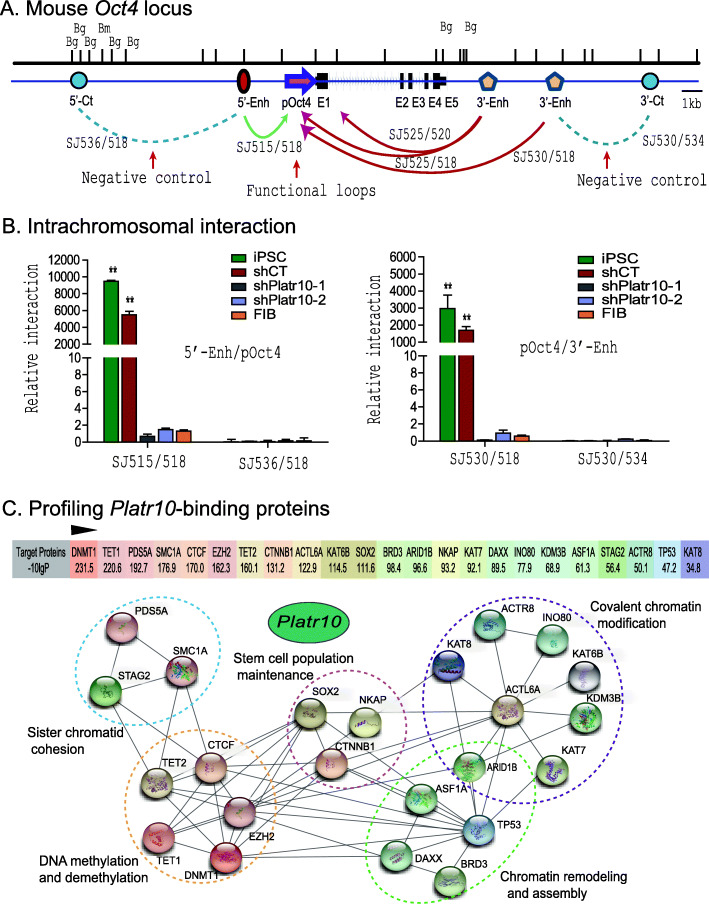
*Platr10* lncRNA is required for the formation of intrachromosomal looping in the *Oct4* locus. **A** Location of 3C primers used to detect the interaction between the *Oct4* promoter and enhancer. Enh, enhancers; pOct4, *Oct4* promoter; 5’-Ct, the 5’ upstream control region of *Oct4*; 3’-Ct, The 3’ downstream control region of *Oct4*; E1-E5, exons; Bm, BamH1; Bg, Bgl2. Arrows: intrachromosomal interactions. **B** Knockdown of *Platr10* abolishes the intrachromosomal interaction loop. shCT, negative control shRNA; shPlatr10, shRNA that targets *Platr10* lncRNA; iPSC, induced pluripotent stem cell; FIB, fibroblasts. Primer sets that detect the presence of looping are marked in red. The 3C interaction was quantitated by qPCR and was standardized over the 3C control *Ercc3* gene. For comparison, the relative 3C interaction was calculated by setting the 5’ or 3’ control as 1. **p < 0.01 as compared with the shPlatr10 treatment and FIB controls. **C** Profiling *Platr10*-binding proteins. The Platr10-binding protein factors were mapped by RNA pulldown MS sequencing using biotin-labeled *Platr10* sense lncRNA in E14 cells. The Platr10 antisense RNA was used as the control. The binding signal was calculated as the protein enrichment ratio (the PEAKS score, −10logP) after adjusting over that of the antisense control. The protein interaction network was constructed using the String database web tool (https://string-db.org/)

We used chromatin conformation capture (3C) [23] to compare intrachromosomal looping between iPSCs and *Platr10* knockdown cells. Cells were fixed with 1% formaldehyde, digested with restriction enzymes *BamH1/BglII*, and then ligated with T4 DNA ligase. After reversal of the crosslinks and DNA purification, the chromatin interaction was detected by specific 3C primers located in the promoter and the enhancers of *Oct4* (Fig. 4A).

As previously reported [9], we detected reprogramming-associated intrachromosomal interaction products in fully reprogrammed iPSCs: the SJ515/SJ518 loop between the 5′ upstream enhance and the promoter, and the SJ530/SJ518 loop between the 3′ enhancer and the promoter (Fig. 4B). However, shRNA knockdown of *Platr10* abolished these intrachromosomal interaction signals and caused the iPSCs to exit from pluripotency. In the control group that was transfected with the shRNA control (shCT), these intrachromosomal loops remained intact. As expected, none of these 3-dimensional interactions were detected in fibroblasts.

We sequenced the 3C products and confirmed the presence of the ligated BamHI or BamHI/BglII sites, which were flanked by the sequences from the promoter and enhancers of *Oct4*, respectively (Fig. S13). These data suggest that *Platr10* is critical for the maintenance of intrachromosomal interactions that are known to be associated with reprogramming and the maintenance of pluripotency [9].

To examine how *Platr10* lncRNA coordinates this pluripotency-associated intrachromosomal looping, we mapped the *Platr10*-binding proteins using RNA pulldown-mass spectrometry (MS) protein sequencing. We found that DNA demethylase TET1 was high on the list of the MS-identified proteins (Fig. 4C), although *Platr10* also interacted with many proteins in other pathways, including chromatin modification and remodeling (Fig. S14A).

### Platr10 recruits TET1 DNA demethylase

CpG DNA demethylation in the *Oct4* promoter is required for nuclear reprogramming. In order to initiate reprogramming, the methylated CpGs in *Oct4* must be demethylated to initiate transcription in somatic cells. TET proteins, a group of Fe(II)/2-oxoglutarate-dependent dioxygenases, have recently been identified as critical factors that induce the oxidation-deamination mechanism underlying active DNA demethylation in mammals [24]. Knockdown of Tet1 induces DNA hypermethylation in the *Nanog* promoter accompanied by defects in self-renewal in ESCs [25]. TET1-initated DNA demethylation is essential for the initiation of reprogramming [26].

**Fig. 5:**
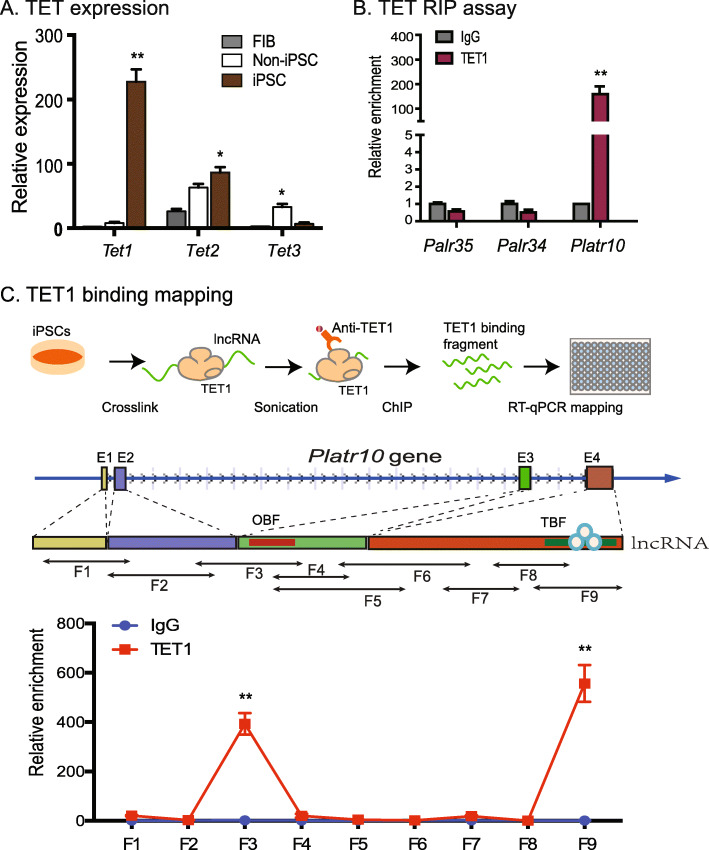
*Platr10* coordinates DNA methylation in the *Oct4* promoter by recruiting TET1 demethylase. **A** Differential expression of TET family genes during reprogramming. Cells were collected at different stages of reprogramming and the expression of the three TET demethylases was measured by RT-PCR. FIB, fibroblasts; non-iPSC, cells that ectopically express OSKM cocktail factors, but fail to complete reprogramming; iPSC, reprogrammed pluripotent stem cells. *p < 0.05, **p < 0.01 as compared with other two groups. **B** Interaction of *Platr10* with TET1 DNA demethylase enzyme by RNA-chromatin immunoprecipitation (RIP). After formaldehyde crosslinking, the TET1-lncRNA chromatin complex was immunoprecipitated with a TET1-specific antibody. After de-crosslinking, the immunoprecipitated RNAs were reverse-transcribed. The TET-interacting lncRNAs were measured by PCR. IgG was used as the antibody control. Input: aliquot DNAs collected during the RIP assay. Note that lncRNA controls *Palr35* and *Palr34* did not interact with TET1, even though these two lncRNAs were also differentially activated in pluripotent reprogramming. **C** Identification of the TET1 binding fragment by RIP mapping. Top panel: Schematic diagram of RIP mapping. iPSC cells were fixed and were subject to a more stringent sonication treatment in order to break the *Platr10* lncRNA regions that are not a part of the TET1 binding site. After immunoprecipitation with a TET1 antibody, the TET1 interacting *Platr10* lncRNA fragments were mapped by quantitative PCR using overlapping primers (middle panel). For comparison, the value of the IgG control was set as 1. **p < 0.01 as compared with other PCR fragments. The F3 and F9 show a strong binding of TET1

We used quantitative PCR to measure the transcript abundance for TET family genes. Among the TET family members, TET1 exhibited differential expression during reprogramming, with high abundance in iPSCs (Fig. 5A). We then asked if the binding of *Platr10* to the *Oct4* promoter would guide this demethylation process in reprogramming. We used an RNA-chromatin immunoprecipitation (RIP) method to pull down TET complexes in iPSCs. The pulled-down RNAs were reverse transcribed and quantitated by PCR using primers for *Platr10*. Using this assay, we detected enrichment of *Platr10* in the TET1 antibody-precipitated complexes (Fig. 5B), suggesting the interaction of the lncRNA with DNA demethylases. No similar interaction was detected in the IgG control group. We also examined if the TET1-pulldown complex contained two control lncRNAs (*Palr35*, *Palr34*) that are also activated in reprogramming. We did not detect either of these two lncRNAs in the TET1 complex (Fig. 5B). We also used a second CLIP assay to validate the *Platr10*-TET1 binding in E14 cells (Fig. S14B). As expected, we found that TET1 bound to *Platr10* in E14 cells. This binding was abolished after shPlatr10 treatment.

We further examined the TET1/*Oct4* interaction in E14 cells (Fig. S15A). For this, we collected E14 cells treated with shPlatr10 and shCT. Using a TET1-specific antibody, we detected the TET1-*Oct4* interaction in E14 shCT control cells. However, treatment of E14 cells with two *Platr10* shRNAs significantly reduced the ChIP signal at the *Oct4* promoter locus (Fig. S15B). These data suggest a critical role of *Platr10* lncRNA in the binding of TET1 to the *Oct4* promoter.

After confirming the role of *Platr10* in the TET1-*Oct4* binding, we used a RIP in situ mapping assay to identify the specific fragment of *Platr10* that interacts with TET1 (Fig. 5C). After crosslinking, iPSCs were lysed and the chromatin fraction was subjected to a longer sonication to fracture unbound RNAs. The TET1-binding RNAs were immunoprecipitated by a TET1 antibody and were reverse transcribed. The TET1-interacting regions were mapped by overlapping PCR (Fig. 5C, top panel). Using this approach, we identified two regions that showed enriched TET1 binding signals (Fig. 5C, bottom panel). The F3 region overlapped with the *Oct4* binding element (OBE) identified from the RAT-seq dataset. The second TET1 binding site F9 was located at the 3′ end of *Platr10*.

### Mapping of the TET1-binding element in Platr10

**Fig. 6:**
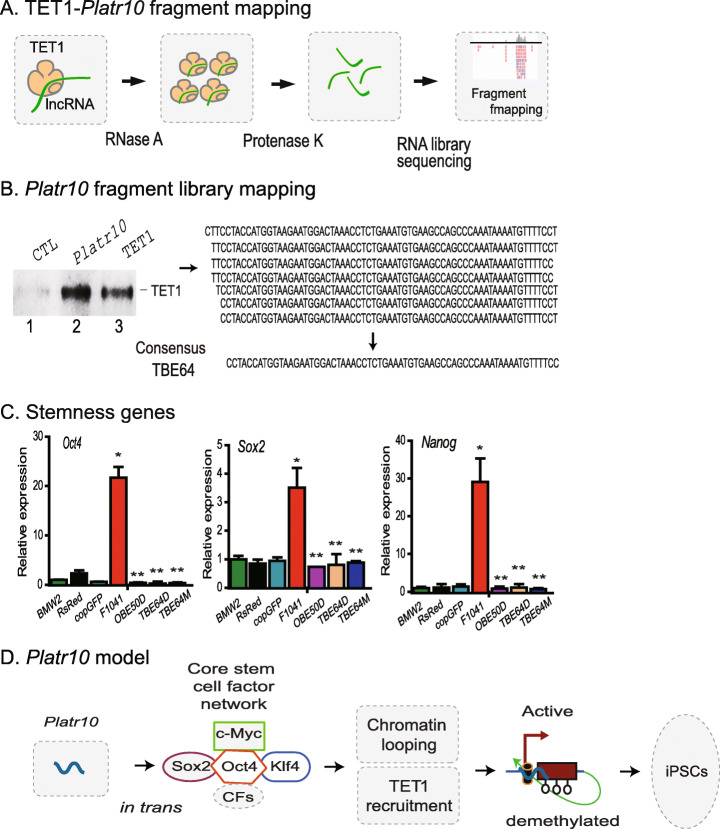
Mutation of the *Oct4* binding element and TET1 binding elements abolishes the function of *Platr10* lncRNA. **A** Diagram of TET1 binding element (TBE) mapping. The biotin-labeled *Platr10* full-length lncRNA was incubated with TET1 recombinant protein. After binding, RNase A was used to remove the free *Platr10* lncRNA fragments. After treatment with proteinase K, the TET1 protected *Platr10* lncRNA fragment was purified and was used for RNA library cloning. DNA sequencing was then performed to map the TET1 binding elements. **B** Identification of the TET1 binding element. Left panel: Western blot detection of the Oscrl8-TET1 interaction. After binding, the *Platr10*-Tet1 complex was pulled-down by streptavidin beads and was subject to Western blot analysis. *Platr10*, biotin-labeled *Platr10* full-length lncRNA; CTL, biotin-labeled *Platr10* antisense lncRNA control; TET1, recombinant protein. Right panel: read sequences of the TET1 binding library. TBE58: consensus TET1 binding element. **C** Requirement of the OBE and TBE elements in *Platr10* lncRNA. Fibroblasts (BMW2) were transfected with lentiviruses carrying full-length *Platr10* or *Platr10* mutants that lack either the OBE or TBE. After puromycin selection, mixed cells were collected for qPCR analyses of the endogenous core stem cell factor genes. *p < 0.01 as compared with controls; **p < 0.01 as compared with the full length *Platr10* lncRNA. The function of *Platr10* was abrogated in the OBE50D, TBE58D, and TBE58M groups. **D** Putative model of *Platr10* in maintaining pluripotency. Open circle under the promoter: unmethylated CpG islands; TET1: DNA demethylases. In un-reprogrammed cells, such as fibroblasts, the *Oct4* promoter is fully methylated and is transcriptionally inactive. During reprogramming, *Platr10* becomes activated. By binding to the core stem cell gene network, *Platr10* orchestrates an intrachromosomal loop, juxtaposing the enhancers close to the promoter. In addition, *Platr10* also recruits TET1 and induces DNA demethylation in the promoter. By activating pluripotency-associated genes, the lncRNA promotes reprogramming and keeps stem cells from differentiation.

We then used an RNase A mapping approach to define the specific TET1 binding element. *Platr10* lncRNA was synthesized in vitro using T7 RNA polymerase and biotin-CTP. The biotin-labeled lncRNA was incubated with TET1 recombinant protein. The mixture was treated with RNase A to remove the unbound lncRNA. The TET1-interacting lncRNA fragment was recovered with streptavidin beads, ligated with short RNA library adaptors, reverse transcribed using an adaptor primer, and cloned into pJet vector for DNA sequencing (Fig. 6A). We mapped the TET1 binding element to the 3′-end (Fig. 6B, right panel), which is identical to the locus as defined by the nuclear RIP assay. Considering the presence of some shorter *Platr10* transcripts from 3′-RACE, a consensus 58 bp fragment was used for further studies. After streptavidin pulldown, the *Platr10*-interacting proteins were eluted and analyzed by Western blot. Using a TET1-specific antibody, we confirmed that *Platr10* specifically interacted with TET1.

We then performed mutation assays to characterize the function of the identified *Platr10* elements. The expression constructs included the full *Platr10* (F1041), deletion of a 50 bp *Oct4* binding element (OBE50D), deletion of a 58 bp TET1 binding element (TBE58D), and mutation of the 58 bp TBE with a random sequence (TBE58M) (Figs. S16A-S16B). To characterize the role of these elements, we stably transfected the expression vectors in fibroblasts. After puromycin selection, stable clones were collected to examine the expression of the core stem cell factor genes *Oct4*, *Sox2*, and *Nanog* (Fig. 6C). Neither the lentiviral DsRed vector control nor the copGFP control altered the activity of the three endogenous stem cell factor genes. However, ectopic expression of the full *Platr10* (F1041) activated these core stem cell factor genes. Deletion of the *Oct4* binding element (OBE50) abrogated *Platr10* activity. Deletion or mutation of the TET1 binding element abolished *Platr10* activity as well.

We also examined the role of *Platr10* in an *Oct4* promoter-luciferase reporter system. The activity of the 4.2 kb *Oct4* promoter was measured using a luciferase kit. As compared with the vector control, the full length *Platr10* (F1041) activated the *Oct4* promoter. In the OBE50D group, deletion of the *Oct4* binding element completely abolished the function of *Platr10*. However, deletion and mutation of the TET1 binding element did not affect the function of *Platr10* in this luciferase co-transfection assay (Fig. S16C). By comparing these in vitro and in vivo data, it appears likely that other regulatory elements may also be required to optimally activate the *Oct4* promoter in vivo. Alternatively, the promoter plasmid DNAs used in the luciferase assay were un-methylated and might not need the participation of TET1 demethylase.

We further examined the role of *Platr10* in a LIF-withdrawal model. Upon LIF withdrawal, E14 cells formed stem cell spheroids slowly when seeded at very low density. However, the full-length *Platr10* was able to partially correct the LIF withdrawal-induced defect, including spheroid formation and *Nanog* expression. This ability, however, was abolished in *Platr10* mutants that lack the 3′-fragment (Fig S17). After *Platr10* knockdown, the *Oct4* promoter became demethylated in parallel with the loss of pluripotency. In addition, expression of *Platr10* partially reversed the altered DNA demethylation in the *Oct4* promoter in LIF-withdrawal E14 cells (Fig. S18). Taken together, these data suggest that both the *Oct4* binding element and the TET1 binding element are critical for the optimal function of *Platr10* lncRNA in activating endogenous core stem cells factor genes.

## Discussion

This study demonstrates a previously undisclosed *trans* role for a lncRNA in the establishment and maintenance of stem cell pluripotency. It has been more than a decade since the discovery that somatic cells can be reprogrammed into pluripotent status by OKSM factors [1]. However, we still know very little about how this reprogramming is initiated. In particular, it is unclear why the majority of cells, even though they express the viral reprogramming factors, fail to convert into pluripotent cells. Recent studies suggest that during reprogramming, chromatin architecture must be appropriately remodeled, so that core stem cell genes occupy preferred territories and are organized in a pluripotency-specific network consisting of specific intra/inter-chromosomal loops [10, 27]. Using CRIST-seq, we profiled the RNAs that interact with the *Oct4* promoter. In conjunction with the RNA-seq data, we identified *Platr10* as a critical pluripotency-associated lncRNA. Loss- and gain-of-function assays confirmed that *Platr10* is essential for optimal maintenance of pluripotency. Knockdown of *Platr10* induces iPSCs to exit from pluripotency. The *Platr10*-deficient iPSCs lose the potential of self-renewal and become differentiated. Overexpression of *Platr10*, on the other hand, triggers the activation of core stem cells factor genes, and enhances the efficiency of pluripotent reprogramming. Most importantly, we demonstrate that *Platr10* is transcribed specifically during reprogramming. In the nucleus, it regulates pluripotency in *trans* by helping to organize a 3-dimensional chromatin interaction network and by recruiting TET1, a DNA demethylase that removes the epigenetic barriers of DNA methylation in the core stem cell gene promoter (Fig. 6D). However, it is not clear if chromatin looping and the methylation status at the *Oct4* locus impact each other. The data from RNA pulldown-MS protein sequencing suggest that *Platr10* binds multiple factors involved in covalent chromatin modification and chromatin remodeling, including SMC1a, CTCF, and Cohesin subunit SA-2 (Stag2). Thus, it is possible that by binding to these chromatin factors, *Platr10* lncRNA may coordinate both the methylation status and chromatin looping at the Oct4 locus. Even more interestingly, the RNA pulldown MS protein sequencing data demonstrate that in addition to TET1, *Platr10* also binds to DNA methyltransferase DNMT1. This finding suggests that by binding to DNMT1, *Platr10* may also regulate stem cell fate by actively coordinating DNA demethylation in many other target genes as identified by RAT-seq. Further studies are needed to address these critical issues.

At the *Oct4* gene locus, for example, the intrachromosomal looping between the promoter and enhancer regulatory regions is specific for pluripotent stem cells. These intrachromosomal interactions have been demonstrated by several technologies, including 3C, Hi-C, and ChIA-PET [28]. This topological structure helps bring distal regulatory elements, like enhancers, into physical proximity with gene target promoters, thereby activating them to initiate cellular reprogramming [9]. In this study, we showed that *Platr10* was enriched in areas containing these regulatory elements, including the promoter, and 5′- and 3′-enhancers, where pluripotent transcription factors such as *Oct4*, *Sox2*, *Nanog*, and other chromatin factors are found [8, 9, 29]. Unlike many transcription factors that bind to target genes within a short stretch of DNA, *Platr10* covers huge areas in the promoter, enhancer, or exons. Our RAT-seq data also show that *Platr10* binds to many targets that do not belong to the accepted classes of stemness genes. Currently, we are not sure whether those target genes are also involved in the establishment and maintenance of pluripotency. It is likely that *Platr10* may have multiple functions in addition to its role of regulating pluripotent genes.

We have demonstrated that *Platr10* is essential for the maintenance of intrachromosomal looping at the *Oct4* locus. Knockdown of *Platr10* abolishes this intrachromosomal loop structure and causes the exit of iPSCs from pluripotency. In addition to *Oct4*, the RAT-seq data also demonstrate that *Platr10* binds to many other pluripotency-associated genes, including *Sox2*, *Klf4*, and *c-Myc*. These four transcription factors have been widely used to reprogram somatic cells. During reprogramming, *Platr10* is actively transcribed and acts in concert with other chromatin factors to coordinate a topological architecture network that is necessary to initiate pluripotency. The Hi-C assay has been used to map the chromatin architecture in human [30] and mouse [31] ESCs. However, the resolution of Hi-C is too low to delineate the promoter-enhancer intrachromosomal loop at the *Oct4* locus. Thus, it will be important to delineate this lncRNA-chromatin interaction in greater detail using a high-resolution approach, like Micro-C, enhanced Hi-C, and Capture-C [32].

Currently, we know very little about how *Paltr10* recognizes its target genes, like *Oct4*. By searching the lncRNA-DNA interaction prediction website (LongTarget: http://lncrna.smu.edu.cn/show/DNATriplex) [33], we could not find an interaction using the base matching mechanism between *Platr10* and the *Oct4* promoter. To explore this issue, we have performed RNA pulldown-MS sequencing for *Platr10* lncRNA. The MS sequencing data show that in addition to TET1, *Platr10* also binds to a number of proteins that are involved in covalent chromatin modification, chromatin remodeling, and chromosome cohesion. Thus, it is possible that this lncRNA-chromatin interaction requires the participation of other chromatin factors, like CTCF and Cohesin. In addition, the RNA pulldown-MS sequencing data suggests that *Platr10* binds to P53. Thus, it is possible that the nuclear *Platr10* may enhance reprogramming, at least partially, by regulating the P53 pathway.

*Platr10* lncRNA also contains a short TBE fragment at its 3′ terminus, which can interact with TET1 demethylase. In fibroblasts, we show that viral expression of the full-length *Platr10* upregulates the core stem cell factor genes *Oct4*, Sox2, and Nanog. When this TBE element was deleted or replaced with a 58 bp random RNA, the *Platr10* mutants completely lose activity. These data suggest that the binding of TET1 demethylase may be an important step to activate core stem cell factor genes during the initiation of pluripotent reprogramming. It should be noted that in addition to its activity to catalyze hydroxylation of 5-methylcytosine to generate 5-hydroxymethylcytosine in DNA, recent studies show that TET1 and TET2 are also critical for RNA hydroxymethylation [34]. The TET family has a C-terminal RNA-binding domain that binds to thousands of RNA targets, including *Platr10*. It is not known if all TET1-interacting RNA molecules have a consensus motif like the *Platr10* TBE.

In addition to the TET family, a recent study by Holmes et al. [35] showed that SOX2 protein also bound to multiple RNA molecules in mECSs. Their formaldehyde cross-linked immunoprecipitation (fRIP) approach mapped 1010 SOX2-binding RNA genes, while the CLIP-seq approach showed 54 enriched genes, with 41 genes overlapping in the two approaches. By analyzing their geo data, it can be shown that *Platr10* appears in both fRIP-seq and CLIP-seq databases. Using RNA pulldown-MS protein sequencing, we also detected the binding of *Platr10* with multiple stemness markers, including SOX2. Thus, it would be interesting to examine if in addition to the role in coordinating intrachromosomal loop at the Oct4 locus, *Platr10* lncRNA may regulate pluripotency through modulating the activity of these pluripotent markers at the post-transcriptional level.

Overall, our work demonstrates a critical role for the lncRNA *Platr10* in both the induction of cell reprogramming and the maintenance of pluripotent status in iPSCs. As a lncRNA, *Platr10* does not encode a known pluripotent factor. However, it regulates pluripotency using unique mechanisms that cannot be fulfilled by pluripotency-associated protein transcription factors. Specifically, it helps maintain a pluripotency-specific chromatin architecture in the *Oct4* promoter. By binding to the *Oct4* locus, it facilitates a functional juxtaposition between the two *Oct4* regulatory regions. With this intrachromosomal loop, the distal enhancer element is moved in close juxtaposition to the promoter, activating this stem cell core factor gene. After binding to *Oct4*, this lncRNA recruits the DNA demethylase TET1, which induces demethylation of the *Oct4* promoter as an essential step to initiate reprogramming. By coordinating the necessary chromatin architecture and DNA demethylation, *Platr10* may play an essential role in initiating pluripotency during reprogramming (Fig. 6D). As *Platr10* is involved in the regulation of pluripotency, it will also be interesting to expand this study by exploring the role of this lncRNA in a knockout mouse model.

## Materials and methods

### Cell lines and cell culture

E14 mouse embryonic stem cells were purchased from ATCC and were cultured in the ESC medium containing KnockOut DMEM, 15% fetal calf serum, l-glutamine, non-essential amino acids, penicillin/streptomycin, and 2-mercaptoethanol and supplemented with LIF. Mouse embryonic fibroblasts (MEFs) were cultured from fetal mice, and MBW2 fibroblast-like cells were cultured from M. spretus-Balb/c F1 mouse bone marrow mesenchymal stem cells [17], and were routinely cultured in Dulbecco’s modified Eagle’s medium (DMEM), supplemented with 10% fetal bovine serum (FBS), 1% non-essential amino acid (NEAA), and 1% antibiotics (penicillin-streptomycin) at 37°C in an atmosphere containing 5% CO_2_.

### Construction of CRIST targeting vectors

CRIST-Seq was performed to identify lncRNAs that bind to the Oct4 and Sox2 promoters (Fig. S1). Specifically, we constructed the Cas9-Oct4 gRNA vector by cloning two Oct4 promoter gRNAs (Fig. S2B) into the lentiCRISPR-EGFP sgRNA 2 vector that contains the catalytically inactive dCas9-FLAG [36, 37]. The U6-gRNA1-T5-H1-gRNA2 T5 cassette was synthesized by joining the H1 promoter with two oligonucleotides that contain the guiding RNA (gRNA) from the *Oct4* promoter (*Oct4*-gRNA1: 5′-GAACATTCAATGGATGTTTT-3′ and *Oct4*-gRNA2: 5′-GTGTGAGGGGATTGGGGCTC-3′), respectively. The expression cassette was inserted downstream of the U6 promoter in the CRIST targeting vector. The same strategy was used to construct the Cas9-*Sox2* gRNA vector. The *Sox2* promoter guiding RNAs include: *Sox2*-gRNA1: 5′-GGGGTTGAGGACACGTGCTG-3′ and *Sox2*-gRNA2: 5′-GAGCCAATATTCCGTAGCAT-3′). A CRIST-Seq control vector was constructed using a Cas9 random gRNA (Cas9-gCT): GTGCGTTGTTAGTACTAATC.

### Cell reprogramming

Reprogramming of mouse fibroblasts into iPSCs was performed by lentiviral *Oct4*-*Sox2*-Klf4-c-Myc-GFP (OSKMN) as previously described [9]. Briefly, fibroblasts in 6-well plates were infected with lentiviruses in the presence of polybrene (8 μg/ml). Three days after infection, the cells were digested and transferred to 100-mm dishes on mitomycin C-inactivated MEF feeder cells. The media were replaced with ES medium (DMEM/F12 supplemented with 20% KSR, 10 ng/ml leukemia inhibitory factor (LIF, Sigma), 10 ng/ml β-FGF (PeproTech), 0.1 mM β-mercaptoethanol, L-glutamine, and 1×10^-4^ M non-essential amino acids [38]. The selected iPSC colon cells had been fully characterized previously by immunostaining stem cell markers, alkaline phosphatase (AP) staining, karyotype analysis, and teratoma formation [9]. The fibroblast-like cells that expressed OSKMN but were not reprogrammed were termed “non-iPSCs” and used in parallel with iPSCs in the study [9, 18].

### CRIST-Seq to map the Oct4-interacting lncRNAs

A CRIST-seq approach was used to map the *Oct4* promoter-interacting lncRNAs as descried [15]. Briefly, iPSCs were transfected with the dCas9-gRNA lentiviruses. After transfection, cells were selected by puromycin and were collected for CRIST immunoprecipitation [39, 40]. To isolate the *Oct4* promoter-interacting lncRNA, cells were cross-linked with 2% formaldehyde and were lysed with cell lysis buffer (10 mM Tris [pH 8.0], 10 mM NaCl, 0.2% NP-40, 1x protease inhibitors). Nuclei were collected by centrifugation and were suspended in 1× reverse transcription buffer in the presence of 0.3% sodium dodecyl sulfate (SDS) and incubated at 37°C for 1 h. Triton X-100 was then added to a final concentration of 1.8% to sequester the residual SDS. An aliquot of nuclei (3×10^6^) was reverse transcribed at 37°C for 30 min in a 20-μl reaction containing 1μl random hexamer, 1μl 10 mM dNTP, 1μl 0.4 mM dCTP -biotin, 1μl RT enzyme, 0.5μl RNase inhibitors, 1μl 0.1M DTT, 4μl 5× cDNA synthesis buffer, 1μl Maxima Reverse Transcriptase, and RNase-free water to 20μl. The reaction was stopped by adding 4μl 0.5M EDTA. After nuclear lysis, the chromatin complex was subjected to sonication for 180 s (10 s on and 10 s off) on ice using a Branson sonicator with a 2-mm microtip at 40% output control and 90% duty cycle settings. The biotin-labeled cDNA/Cas9 complex was immunoprecipitated with anti-FLAG antibodies (#MA1-91878, Thermo Fisher, IL, or #F7425, Sigma, MO). The DNAs were released after cross-linking reversal and proteinase K treatment and were precipitated with ethanol. The biotin-labeled cDNAs were further purified from genomic DNAs with M-280 streptavidin beads (Invitrogen, CA). After purification, the *Oct4* promoter-interacting cDNAs were quantitated by PCR using target gene primers (Table S1).

For Illumina cDNA sequencing, the second-strand cDNA was synthesized using a Stratagene cDNA Synthesis kit (Agilent Technologies, CA). The double-strand cDNAs were digested by Dpn I for library construction by ligating with the NEBNext adaptors (NEBNext® ChIP-Seq Library Prep Master Mix Set for Illumina). Triplicate library DNAs were sent to Shanghai Biotechnology (Shanghai) for Illumina sequencing.

For CRIST-Seq control, we performed a CRIST assay using a random gRNA (gCT) and constructed the control library for sequencing using the same protocol. An anti-IgG antibody was used as the background control for immunoprecipitation [18].

### CRIST-Seq data analyses

After CRIST sequencing, the adapter sequences were removed from the raw data using Illumina annotated adapter sequences with parameter ILLUMINACLIP: 2:30:10 and the low-quality data were filtered using Fastx software (http://hannonlab.cshl.edu/fastx_toolkit/index.html). After filtering, clean reads were mapped to the mouse genome (genome version: mm10, GRCm38.p4) for mRNAs and lncRNAs using TopHat (version:2..0.9) software [41]. The mapped RNA reads were quantitated using Cuffling (version:2.1.1). Gene counts were normalized to the values of fragments per kilobase of transcript per million fragments mapped (FPKM). The resulting coverage tracks (bedgraph file) were visualized in UCSC genome browser. The peak was called and annotated with RIPSeeker. The called peaks that overlapped with the IgG control enriched regions were removed. To define the specific binding of RNAs, the CRIST-Seq signal intensities were further normalized over that of the non-targeting Cas9 gCT control with the DiffBind package using parameters of fold change difference ≥2 and *p* value < 0.05, with false discovery rate (FDR) <0.1. The adjusted CrIST-Seq data were then used for mapping the Oct4 and Sox2 RNA interactions [18].

### RNA-Seq to identify differentially expressed lncRNAs in reprogramming

Total RNA was isolated from fibroblasts and iPSCs [9] using TRIzol (Invitrogen, Carlsbad, CA). The indexed libraries were prepared using Illumina’s TruSeq RNA Sample Prep Kit v2. Paired-end sequencing in triplicate was performed by Shanghai Biotechnology (Shanghai, PRC) using a HiSeq4000 (Illumina). The RNA-Seq yielded 145 million raw reads for iPSC and 148 million raw reads for fibroblasts. After Seqtk filtering, a total of 120 million clean reads for fibroblasts and 124 million clean reads for iPSCs were mapped to the mouse genome (genome version: mm10, GRCm38.p4 (ftp://ftp.ensembl.org/pub/release-83/fasta/mus_musculus/dna/Mus_musculus.GRCm38.dna.primary_assembly.fa.gz) for mRNAs and lncRNAs using the STAR software. Gene counts were normalized to the values of reads per kilobase of transcript per million mapped reads (RPKM). Cuffdiff was used to calculate the differentially expressed RNAs when the fold-change was > 2 and *p* < 0.05 with an unpaired two-sided *t* test [18].

### Integration of RNA-Seq and CRIST-Seq data

Reprogramming-associated RNA candidates were identified by RNA-Seq using a cutoff threshold of >2-fold, *p*<0.05. CRIST-Seq RNAs were selected by peak enrichment FPKM>50 as a cut-off threshold after adjusting over the IgG control and Cas9-gCT control. The RNA-Seq data were merged with the *Oct4* and *Sox2* CRIST-Seq data using VENN program (http://bioinformatics.psb.ugent.be/webtools/Venn/). Venn diagrams were used to visualize the overlap RNAs between the datasets. The overlapping RNAs identified by the datasets were chosen for further functional characterization.

### Characterization of Platr10 lncRNA

During the cloning of full length *Platr10* cDNA, our PCR products indicated that the lncRNA underwent alternative splicing. Therefore, we characterized *Platr10* lncRNA by cDNA 5′- and 3′-RACE following the method as previously described [21]. For the 3′-end racing, total iPSC RNAs were reverse transcribed using Maxima Reverse Transcriptase with a poly T primer: SJ773 5′-CTGCGTAATACGACTCACTATAGGAGACAGGCTCGAGTTTTTTTTTTTTTTTTTT-3′. The first racing PCR was performed using 3′-RACE primer SJ771: 5′-CTGCGTAATACGACTCACTATAGG-3′ and a 5′-*Platr10*-specific primer SJ938: 5′-CTGTTGAGC CAGGCAGCTG-3′. The PCR DNAs were diluted 500-fold and were used for the second nested PCR using 3′-RACE primer SJ772: 5′-GACTCACTATAGGAGACAGGCTCGA-3′ and a 5′-*Platr10*-specific primer SJ937: 5′-AGCTGGAGGAAGTGTGTCAC-3′. The racing PCR bands were cut and cloned into pJet vector for sequencing.

For the 5′-racing, the iPSC RNAs were reverse-transcribed using Maxima Reverse Transcriptase with random hexamer oligonucleotides for 30 min. The poly G primer SJ774: 5′-CCAGATTCAGGACTGTCGACATCGAATTCGGGG-3′ was added into the mixture and the reaction was continued for another 30 min. After 10-fold dilution, cDNAs were amplified with 5′-adapter RACE primer SJ775: 5′-GATTCAGGACTGTCGACATCGA-3′ and *Platr10*-specific primer SJ935: 5′-TCATGTCCGGGTTCGAGCCT-3′. The PCR bands were cloned into pJet vector for sequencing.

### RAT-Seq assay to map the genome-wide interacting target genes for Platr10

A RAT assay was modified to map the genome-wide interacting target genes for *Platr10* [42]. Specifically, cells were cross-linked with 2% formaldehyde and lysed with cell lysis buffer (10 mM Tris [pH 8.0], 10 mM NaCl, 0.2% NP-40, 1x protease inhibitors). Nuclei were suspended in 1× reverse transcription buffer in the presence of 0.3% sodium dodecyl sulfate (SDS) and incubated at 37°C for 1 h. Triton X-100 was then added to a final concentration of 1.8% to sequester the SDS. Gene strand-specific reverse transcription was performed using four *Platr10*-specific complementary primers in the presence of biotin-dCTP. After 50 min of reverse transcription of *Platr10* lncRNA with Maxima Reverse Transcriptase (Thermo Fisher Scientific, CA) at 62C, the reaction was stopped by adding 4μl 0.5M EDTA. After nuclear lysis, the chromatin complex was subjected to sonication for 180 s (10 s on and 10 s off) on ice with a Branson sonicator with a 2-mm microtip at 40% output control and 90% duty cycle settings. The biotin-labeled cDNA/chromatin DNA complex was pulled down with biotin-streptavidin magic beads (Invitrogen, CA). After reversing the cross-links and washing with 10 mg/ml proteinase K at 65°C overnight and treatment with 0.4 μg/ml RNase A for 30 min at 37°C, the genomic DNA that interacted with *Platr10* was extracted and digested by Dpn I, and ligated with the NEBNext adaptors (NEBNext® ChIP-Seq Library Prep Master Mix Set for Illumina) to construct the library. Triplicate library DNAs were sent to Shanghai Biotechnology for Illumina sequencing.

For RAT-Seq control, we performed a RAT assay by replacing *Platr10* complementary primers with random primers and constructed a control library for sequencing using the same protocol.

### RAT-Seq data analyses

After RAT sequencing, the low-quality reads were filtered using Fastx (version:0.0.13) software (http://hannonlab.cshl.edu/fastx_toolkit/index.html). Clean reads were mapped to the mouse genome (genome version: mm10) using the Bowtie (version:0.12.8) software with default parameters. Enriched regions of the genome were identified by comparing the RAT-Seq peaks to input samples using MACS2 (version:2.1.1), and *q* value of 0.05 was used as the initial cutoff threshold to minimize peak caller bias. The upstream 2k of the transcription start sites and the downstream 5k of the transcription termination region were defined as the gene regions. The significant GO terms of biological processes with a *p* value < 0.05 were selected. We also used the MEME suite [43] for the discovery and analysis of the peaks’ sequence motifs. The resulting coverage tracks (bedgraph file) were visualized in the UCSC genome browser. To reduce the background, the RAT-Seq data were further normalized over the peaks of the control RAT-Seq data that were generated by using random oligonucleotide primers in the RAT assay. Differential binding analysis was performed with the DiffBind package using parameters of fold-change difference >2 and *p* value < 0.05, with false discovery rate (FDR) <0.1. The adjusted RAT-Seq data were used for mapping the lncRNA target gene interaction network [44, 45].

### RT-PCR

Total RNA was extracted by TRIzol reagent (Sigma, MO) from cells and stored at −80°C. RT-PCR reaction was performed with a Bio-Rad Thermol Cycler. The target amplification was performed by PCR of 1 cycle at 95°C for 5 min; 33 cycles at 95°C for 20s, 62°C for 15s, and 72°C for 15 s; and 1 cycle at 72 °C for 10 min.

Quantitative real-time PCR was performed using SYBR GREEN PCR Master (Applied Biosystems, USA) as previously described [9, 46]. The threshold cycle (Ct) values of target genes were assessed by quantitative PCR in triplicate using a sequence detector (ABI Prism 7900HT; Applied Biosystems) and were normalized over the Ct of the β-ACTIN control.

### RNA fish

RNA FISH was performed by a modification of the method as previously described [47]. The RNA FISH probe was prepared as antisense single-strand DNA (ssDNA) by asymmetric PCR. Briefly, single-strand DNA probes were synthesized by Klen-Taq DNA polymerase using *Platr10* cDNA as the template, PCR primer JH4022: 5′-CACTGCTGGTTTGGAGCTCCAT-3′ and JH4023 5′-TGGGACAGTCTCTGGATGGCCT-3′ in a ratio of 1:50, with dig labeling dNTP MIX (Roche:11277065910). PCR probe products were purified by electrophoresis on 2% agarose gel and eluted in 20 μl TE buffer. For hybridization, 0.1 μg ssDNA probe and 10 μg salmon sperm DNA (Boehringer, Meylan, France) were precipitated with ethanol and suspended in 10 μl RNA hybridization buffer (2xSSC, 10% dextran sulfate, 0.2mg/mL BSA (Invitrogen, CA), and 2mM VCR, 10% formamide). Slides were counterstained with DAPI and FISH signals. Images were captured and merged to confirm subcellular localization.

### Knockdown of Platr10 by shRNA lentiviruses

*Platr10* was knocked down by two separate shRNA lentiviruses. The shRNA vector was constructed by cloning four shRNAs into a pGreenPuro vector (#SI505A-1, SBI, CA) under the control of the H1 and U6 promoters. Vector 1 (shPaltr10-1) contained shRNA #1: 5′-TTCTGTGTATCTGTTGAGCCAG-3′ and #3: 5′-CTGCCAGCATCTGACTAAGATA-3′. Vector 2 (shPlatr10-2) contained shRNA #2: 5′-CCTGCTGCCTGTCAATCCAAAT-3′, and #4: 5′-CCTGCTGCCTGTCAATCCAAAT-3′ (Table S1). The promoter-shRNA cassettes were ligated by PCR and were ligated into the EcoR1/BamH1 site in the pGreenPuro vector. The copGFP reporter in the vector was used to track the lentiviral transfection in iPSCs. Two random shRNAs (5′-GCAGCAACTGGACACGTGATCTTAA-3′ and 5′-TGAAATGTACTGCGCGTGGAGACTA-3) were cloned in the same vector as the assay control (shCT).

### Genomic deletion of Platr10 by CRISPR Cas9 editing

The *Platr10* gene was deleted in E14 cells using CRISPR Cas9 editing following the protocol in our lab [48]. Briefly, two Cas9 targeting vectors were constructed based on the dual SpCas9-NmCas9 cassette reported by Bolukbasi et al. [20]. Four gRNAs were designed from the *Platr10* promoter and 3′-downstream region, respectively (Table S1). As the control, random Cas9 gRNAs were used to construct the Cas9 gCT vector. For homologous recombination, a donor vector was constructed to carry two *Platr10* arm. The copGFP/Puro cassette was used for the positive selection, and the TK for the negative selection. After electroporation, E14 cells were selected by puromycin and ganciclovir, respectively, as previously described [49]. The copGFP-positive cells were collected, and homozygous deletion of *Platr10* was confirmed by PCR and DNA sequencing.

### Platr10 promotes DOX-OSKM reprogramming

*Platr10* cDNA was cloned into pCMV-DsRed-Puro vector, and lentiviruses were packaged in 293 cells. Control lentiviruses carried the pCMV-DsRed-Puro empty vector (Vector) and the pCMV-DsRed-Puro CTL (800 bp random sequence). OG2 MEFs were first transfected with the *Platr10* and control lentiviruses and were selected by puromycin. MEFs were reprogrammed following the method as described [19]. Briefly, 15,000 lentivirus-transfected MEFs were seeded in 12-well plates and were cultured in KSR iPS medium containing 2 μg/ml doxycycline (DOX). The medium was changed every other day. The iPSC colonies were immunostained with Rabbit anti-NANOG Antibody (A300-397A, Bethyl, 1:500 dilution) and alkaline phosphatase (AP) kit (AP100R-1, System Biosciences).

### Immunohistochemical staining of stem cell markers

Immunofluorescent staining was used to examine stem cell markers in iPSC colonies [50]. Briefly, cells were fixed by 4% paraformaldehyde/PBS for 10–15 min and rinsed with PBS, then permeabilized and blocked with 0.1% Triton X-100/PBS containing 3% BSA for 30 min. After incubation with primary antibodies for 1 h at room temperature or overnight at 4C, the samples were washed three times with PBS and then incubated with secondary antibody for 1 h. The following antibodies were used in the immunostaining: rabbit anti-NANOG (1:100 dilution, Santa Cruz) and rabbit anti-OCT4 (1:100 dilution, Millipore). The cell samples were subsequently incubated with Cy3- or Alexa Fluor 488-labeled secondary antibodies for 1 h. After washing three times with PBS, samples were counterstained with Hoechst 33258 (Invitrogen). Alternatively, the pluripotency of stem cells was examined by Fluorescent Mouse ES/iPS Cell Characterization kit (Cat.#SCR077, Millipore, MA) following the protocol provided by the manufacturer. Fluorescence images were acquired with a Zeiss AxioCam Camera.

### Embryoid body differentiation

For embryoid body (EB) formation, iPSCs or E14 cells were dispersed by collagenase IV (Invitrogen), and cell clumps were transferred to 60-mm dishes in ES medium without LIF. After being maintained in floating culture for 3 days, EB were seeded in 0.1% gelatin-coated 6-well plates in DMEM/F12 containing 20% FBS for spontaneous differentiation. Cells were collected at different time points and used for gene expression analysis using quantitative PCR.

### Platr10 RNA pull-down mass spectrometry analysis

*Platr10*-binding proteins were mapped by RNA pull-down mass spectrometry sequencing (Shanghai Jixue Technology Co., Shanghai). Sense biotin-*Platr10* lncRNA was synthesized by T7 RNA polymerase using Biotin RNA Labeling Mix, treated with RNase-free DNase I, and purified with RNeasy Mini Kit (Qiagen). Antisense bitoin-*Platr10* RNA was used as the control. Both strands were used to pull-down target proteins. Differential protein bands were excised and analyzed by mass spectrometry. Fragment sequences from MS were performed using PEAKS 7 at a false discovery rate (FDR) threshold of <5%. *Platr10*-bindnig proteins were mapped after adjusting over the signal of the antisense RNA as the PEAKS score (−10logP).

### RIP mapping of the TET1 binding element

A lncRNA-affinity binding precipitation assay (RIP) [51] was performed to examine the binding of TET proteins with *Platr10* lncRNA. Briefly, iPSCs were fixed with 1% formaldehyde, treated with DNase I, and sonicated using a Branson sonicator. Sonicated samples were immunoprecipitated with antibodies against TET1 and TET3 (Abcam, MA). IgG was used as the experimental control. The precipitated RNA was released, and cDNA was synthesized. After proteinase K treatment, the TET-binding cDNAs were detected by PCR.

To map the TET1 binding fragment, a longer sonication was used by extension to 30 min. The samples immunoprecipitated by the IgG antibody were used as the RIP control. The RIP samples were quantitated by overlapping qPCR primers (Table S1). The Ct values were normalized over the input and compared with the IgG control. It was assumed that the TET1 binding site would be protected from sonication and would have a greater chance of being amplified by qPCR mapping.

### Characterization of the TET1 binding element

An RNase A protection assay [52] was used to identify the specific *Platr10* lncRNA sequence that interacts with TET1. The full length *Platr10* lncRNA was synthesized using HiScribe^TM^ T7 Quick High Yield RNA Synthesis Kit (NEB, MA) with biotin-CTP. The biotin-*Platr10* lncRNA was purified using Streptavidin Magnetic Beads (Pierce™ Magnetic RNA-Protein Pull-Down Kit, Thermo, USA) following the protocol provided by the manufacturer. For TET1 binding mapping, biotin-*Platr10* lncRNA (5 ng/μl) was incubated with TET1 recombinant protein (150 nM) in 150 μl Buffer (20 mM HEPES pH 7.5, 50 mM NaCl, 2 mM MgCl2, and 2 mM DTT) at 22°C for 10 min. After binding, 2 ng/μl RNase A was added to the reaction mixture and was incubated at 22°C for 5 min to remove the RNAs that were unbound or free from the TET1 binding. After RNase treatment, 500 μl TRIzol (Thermo, MA) was added to quench the digestion, and RNAs were purified following the manufacturer’s protocol. An RNA library was prepared using NEBNext Multiplex Small RNA Library Prep Set for Illumina (E7300, NEB, MA). RNA library products were purified by gel extraction and were cloned into a pJet vector for DNA sequencing. The sequencing reads were aligned with the *Platr10* lncRNA sequence to locate the consensus TET1 binding motif. The RNA motif structure of TBE was obtained by submitting the 58 bp consensus sequence to the RNA structure prediction website: https://rna.urmc.rochester.edu/RNAstructureWeb/Servers/Predict1/ResultsPages/20180916.191929-72078a8d/Results.html.

### Quantitation of intrachromosomal looping by chromosome conformation capture (3C)

The 3C assay was performed to determine long range intrachromosomal interactions [53]. Briefly, fibroblasts and iPSCs were cross-linked with 2% formaldehyde and lysed with cell lysis buffer. An aliquot of nuclei (2×10^6^) was digested with 800 U *BamH* I/*Bgl* II at 37°C overnight. Chromatin DNA was diluted with NEB ligation buffer and ligated with 4000 U of T4 DNA ligase. After reversing the cross-links, DNA was purified and used for PCR amplification using primers that are derived from different regions of the *Oct4* locus. The 3C PCR products were cloned and sequenced to validate the intrachromosomal interaction by checking for the presence of the *BamH* I/*Bgl* II ligation site. Each intrachromosomal loop had its own negative control site. The 3C interaction was quantitated by qPCR and was standardized over the 3C ligation control for the housekeeping gene *Ercc3*. For comparison, the relative 3C interaction was calculated by setting the 5′ or 3′ control as 1.

### DNA methylation analysis in the gene promoter

Genomic DNAs were extracted from fibroblasts that express the full length *Platr10* and its mutants and were treated with sodium bisulfate. Methylation PCR was performed, and PCR DNAs were cloned into pJet vector for sequencing. After treatment with sodium bisulfate, unmethylated cytosines were converted to uracils, which can be distinguished from the methylated cytosines by sequencing.

### Mutation of Platr10 lncRNA

To define the function of *Platr10* lncRNA, we constructed a series of lentiviral vectors that express the full length *Platr10* and its mutants. The full-length *Platr10* was amplified from iPSC cDNAs using primers JH4399 5′-CGCGTCGATATCCTCGAGGGAGCCTACACGTGGTCACCTG-3′ and JH6209 5′-GAATCGAAGAATTCGCATGGCAGCATGAAGGCAGACAT-3′. The OBE50D vector was synthesized by overlapping PCR using primers JH4399 and JH6212 5′-GGAAGAATCACAAGTCTGTGTTTCCTTCTCCGGTATGAAT-3′ for PCR fragment 1, and JH6213 5′-AGGAAACACAGACTTGTGATTCTTCCCATCTGCAGA-3 and JH6209 for PCR fragment 2. To construct TBE58D vector, the *Platr10* insert was amplified by primers JH4399 and JH6214 5′-GAATCGAAGAATTCGTCGACAGGAAAACATTTTATTTGGGCTGGC-3′. The TBE58M vector was constructed by a two-step PCR using primers JH4399 with primer JH6210 5′-CAGTATCTGATCTACTATGTAGCATAAGATCATCAGTCCAGCATGGCAGCATGAAGGCAGACAT-3′ and JH6211 5′-GAATCGAAGAATTCCAGAGGATCATCCCACTTCAGTATCTGATCTACTATGTAGCAT-3. All PCR inserts were cloned into a lenti-DsRed vector by EcoRV/EcoR1.

The lentiviruses were packaged in 293T and were used to transfect fibroblasts. After transfection, cells were treated with puromycin, and stable cells were used for the measurement of gene expression of Oct4, Sox2, and Nanog.

### The Oct4 promoter-luciferase assay

The function of *Platr10* in activating the Oct4 promoter was first examined in 293T cells by using a dual-luciferase reporter assay. A 3.9 kb genomic DNA fragment covering the Oct4 promoter and part of the exon 1 sequence was amplified by PCR using primers: SJ559 5′-TATCGATAGGTACCGTCTGTGAGGAGGTGGCTGAACT-3′ and SJ560 5′-atcgcagatCTCGAGCTCCTCGGGAGTTGGTTCCAC-3′. The DNA fragment was cloned into pGL3 vector by Kpn1/Xho1.

For the luciferase assay, cells were seeded at a density of 5 × 10^4^ cells/well in 96-well plates. The lentiviral expression vectors, including *Platr10* full length lncRNAs (F947), OBE50D, TBE58D, and TBE58M were co-transfected with a pOct4-luciferase plasmid and Renilla luciferase control plasmid (Promega) using Lipofectamine 3000 (Invitrogen, CA). The empty lentiviral vector was used as the control. Forty-eight hours after transfection, firefly and Renilla luciferase activities were measured with the dual-luciferase reporter system (Promega) using a luminometer (Turner Biosytem, CA). The relative activity of the Oct4 promoter was calculated by standardized setting the untreated control cells as 1. All luciferase assays were repeated three times.

### Statistical analysis

The data were expressed as mean ± SD and were analyzed using SPSS software (version 16.0; SPSS, Inc., IL). Student’s *t* test or one-way ANOVA (Bonferroni test) was used to compare statistical differences for variables among treatment groups. Results were considered statistically significant at *p*<0.05.

## Supplementary Information

**Additional file 1:.** Supplementary files include the Methods and additional Extended Data.

**Additional file 2:.** Review history
