# Author Correction: Chromatin lncRNA *Platr10* controls stem cell pluripotency by coordinating an intrachromosomal regulatory network

**DOI:** 10.1186/s13059-021-02487-9

**Published:** 2021-09-20

**Authors:** Zhonghua Du, Xue Wen, Yichen Wang, Lin Jia, Shilin Zhang, Yudi Liu, Lei Zhou, Hui Li, Wang Yang, Cong Wang, Jingcheng Chen, Yajing Hao, Daniela Salgado Figueroa, Huiling Chen, Dan Li, Naifei Chen, Ilkay Celik, Yanbo Zhu, Zi Yan, Changhao Fu, Shanshan Liu, Benzheng Jiao, Zhuo Wang, Hui Zhang, Günhan Gülsoy, Jianjun Luo, Baoming Qin, Sujun Gao, Philipp Kapranov, Miguel A. Esteban, Songling Zhang, Wei Li, Ferhat Ay, Runsheng Chen, Andrew R. Hoffman, Jiuwei Cui, Ji-Fan Hu

**Affiliations:** 1 Key Laboratory of Organ Regeneration and Transplantation of Ministry of Education, Stem Cell and Cancer Center, First Hospital, Jilin University, Changchun, Jilin 130061 People’s Republic of China; 2 Stanford University Medical School, VA Palo Alto Health Care System, Palo Alto, CA 94304 USA; 3 CAS Key Laboratory of RNA Biology, Institute of Biophysics, Chinese Academy of Sciences, Beijing, 100101 People’s Republic of China; 4 La Jolla Institute for Allergy and Immunology, La Jolla, California, 92037 USA; 5 Department of Endocrinology, Xiangya Hospital, Central South University, Changsha, Hunan People’s Republic of China; 6 Guangzhou Institutes of Biomedicine and Health, Chinese Academy of Sciences, Guangzhou, Guangdong 510530 People’s Republic of China; 7 Google Inc., Mountain View, CA 94043 USA; 8 Institute of Genomics, School of Biomedical Sciences, Huaqiao University, Xiamen, 361021 People’s Republic of China

**Correction to: Genome Biol 22, 233 (2021)**

**https://doi.org/10.1186/s13059-021-02444-6**

Following publication of the original article [1], the authors identified an error in the author group. The author, Daniela Salgado Figueroa, had erroneously been omitted from the group. The author group has been updated in this correction article and the original article [1] has been corrected.
