# Supplementary material for: Chromatin lncRNA Platr10 controls stem cell pluripotency by coordinating an intrachromosomal regulatory network
Source: Genome Biol. 2021 Aug 19;22:233. doi: 10.1186/s13059-021-02444-6 (PMC8375132; doi:10.1186/s13059-021-02444-6)
Supplement: Supplementary file 1 — Additional file 1:. Supplementary files include the Methods and additional Extended Data. [file 13059_2021_2444_MOESM1_ESM.pdf]

**Chromatin lncRNA *Platr10* controls stem cell pluripotency by coordinating an intrachromosomal regulatory network**

Zhonghua Du[1,2*], Xue Wen[1*], Yichen Wang[1,2*], Lin Jia[1,2*], Shilin Zhang[1,2], Yudi Liu[1,2], Lei Zhou[1,2], Hui Li[1], Wang Yang[1], Cong Wang[1], Jingcheng Chen[1], Yajing Hao[3], Huiling Chen[2,4], Dan li[1], Naifei Chen[1], Ilkay Celik[2], Yanbo Zhu[1], Zi Yang[1,2], Changhao Fu[1,2], Shanshan Liu[1,2], Benzheng Jiao[1,2], Zhuo Wang[1,2], Hui Zhang[7], Günhan Gülsoy[5], Jianjun Luo[3], Baoming Qin[7], Sujun Gao[1], Philipp Kapranov[6], Miguel A. Esteban[7], Songling Zhang[1], Wei Li[1], Ferhat Ay[8], Runsheng Chen[3], Andrew R. Hoffman[2#], Jiuwei Cui[1#], Ji-Fan Hu[1,2#]

## SUPPLEMENTAL TABLES

### Table S1. Oligonucleotide primers used for PCR

| ID | Oligo Name | Oligo sequence | Product size |
|---|---|---|---|
| ***RT-PCR*** | | | |
| *Platr10* | SJ229 | CACTGCTGGTTTGGAGCTCCAT | 121bp |
| | SJ230 | TGGGACAGTCTCTGGATGGCCT | |
| Oct4 | JH116 | CAATGCCGTGAAGTTGGAGAAG | 179bp |
| | JH117 | GGCTGAACACCTTTCCAAAGAGA | |
| Sox2 | JH118 | GGTTACCTCTTCCTCCCACTCCAG | 193bp |
| | JH119 | TCACATGTGCGACAGGGGCAG | |
| Nanog | JH120 | TCTCCTCCATTCTGAACCTGAGC | 150bp |
| | JH121 | TGCTGGGATACTCCACTGGTGCT | |
| Palr34 | JH4413 | GATTGACTAACCAGGGTGGAC | 132bp |
| | JH4414 | CACTCGAGGGAGTCAATGCAG | |
| Palr33 | JH4119 | GAGATGGTGGCTAAACCAGG | 103bp |
| | JH4120 | GAGGCACTGGAGACCATGATG | |
| Palr35 | JH4123 | CAAGATGGGCAGCCTAGTTC | 123bp |
| | JH4124 | GGCATTTGGGGTGAGCTAGGGC | |
| β-Actin | J880 | CAGGTCATCACCATTGGCAATGAGC | 135bp |
| | J881 | CGGATGTCCACGTCACACTTCATGA | |
| U2 | JH1055 | ATCTGTTCTTATCAGTTTAATATCTG | 151bp |
| | JH1056 | GGGTGCACCGTTCCTGGAGGTAC | |
| Tet1 | JH6031 | GAACAGCCAYCAGATCTGTAAG | 170bp |
| | JH6032 | CTGAYTTGGGGCCATTTACTG | |
| Tet2 | JH6033 | GTCCTYATGTGGCAGCTATTAG | 129bp |
| | JH6034 | TAGCAATAGGACATCCCTGAG | |
| Tet3 | JH6035 | CYAAGAGTCTGCTGGACACAC | 154bp |
| | JH6036 | TCCTCCATGAGTTCCCGGATA | |
| ***RAT Oct4 binding*** | | | |
| 5’-CT | SJ476-JH4348 | CTGAGTCCTCTGCAAGATGC | 137bp |
| | SJ477-JH4349 | CCAAGGCACCTGCCTAGGATT | |
| pOct4 | SJ484-J648 | CAGAGGATGGCTGAGTGGGCTGTA | 123bp |
| | SJ485-JH4354 | CACCCCTGCCTTGGGTCACCG | |
| Off-target | JH5877 | AGCCATCCTGTCCTCCGCCTG | 128bp |
| | JH5878 | CTGCACGGAAGGTCACGATG | |

| | |
|---|---|
| ***Platr10 shRNAs*** | |
| 1 | TTCTGTGTATCTGTTGAGCCAGGCA |
| 2 | CCTGCTGCCTGTCAATCCAAATGTA |
| 3 | CTGCCAGCATCTGACTAAGATAGAT |
| 4 | TAGTATGGCTGTCCTCGGAGAGGCT |
| Control (shCT) | GCAGCAACTGGACACGTGATCTTAA |
| | TGAAATGTACTGCGCGTGGAGACTA |
| ***CRIST Cas9 gRNA*** | |
| Oct4-1 | GAACATTCAATGGATGTTTT |
| Oct4-2 | GTGTGAGGGGATTGGGGCTC |
| Sox2-1 | GGGGTTGAGGACACGTGCTG |
| Sox2-2 | GAGCCAATATTCCGTAGCAT |
| Control (gCT) | GAAGTGGGATGATCCTCTGA |
| *CRIST PCR* | |
| SJ516 | CCGCATCTGGCTTCCCAGGATAC |
| SJ517 | CGTTAACTTGCCACAAACCACCTG |
| SJ484 | CAGAGGATGGCTGAGTGGGCTGTA |
| SJ485 | CACCCCTGCCTTGGGTCACCG |
| JH5683 | CAGCTCTCAGGAGGTTCCAG |
| JH5684 | CACTTCCACCAACTAGGAACT |
| ***3C primers*** | |
| *Oct4 functional loop* | |
| SJ518 | GCAGACAGGCACTCTGAGGGC |
| SJ536 | TGCAGGTCTTGGGCACCCCTG |
| SJ515 | ACCATCTCTGGCTGGGGACGTG |
| SJ530 | AGGCTCCGGGTGGAGAAAGCTG |
| SJ534 | GTCTCATGCAGCCCAGGCTGAC |
| SJ525 | ACTGACTGCTCTGCCAGAGGTC |
| SJ520 | GGACACCTGGCTTCAGACTTCG |
| *Igf2/H19 positive control* | |
| SJ1175 | GAAAGCTTCCTTGGTCAGGCTA |
| SJ1202 | TCTGGAAGGGGTGGCAGGGCT |
| SJ1196 | ACCAGGTGAGACCAGCATGAT |
| *Ercc3 ligation control* | |
| JH6286 | TGGAGCAGTGGAAAGCCCAGT |
| JH6282 | CTTCATAAGTGTCTTAGCAGAGCT |

***RAT primers***

| | |
|---|---|
| *Platr10* | |
| JH4397R | CATGATGCTGGAGAGGTAGCT |
| JH4398R | AGAGGGAATCTAGGCAGGTAG |
| JH4023R | TGGGACAGTCTCTGGATGGCCT |
| SJ225R | GACATGATGCTGGAGAGGTAGC |
| *Palr34* | |
| JH4122R | GCCTCAGAAGCTTAAAAGCC |
| JH4164R | GTAGATTTATCAGGGCAGCACAAG |
| JH4414R | CACTCGAGGGAGTCAATGCAG |
| *Palr35* | |
| JH4124R | GGCATTTGGGGTGAGCTAGGGC |
| JH4175R | GTTAACGCTGGTGCCGAGTG |
| JH4176R | GGTACTGCTCCATTTTGCGCATC |
| JH4177R | CATGCACCCAGGTTAGTTGAC |
| *RAT control* | |
| JH5849 | ATGGACTGATGATCTTATGC |
| JH5850 | TACATAGTAGATCAGATACT |

***TBE mapping***

| | |
|---|---|
| F1 JH6155 | CACGTGGTCACCTGAAGCTG |
| JH6156 | TCATGTCCGGGTTCGAGCCT |
| F2 JH6157 | TCCGAGATGCCTCCCAGGCT |
| JH6158 | ATGGAGCTCCAAACCAGCAGTGT |
| F3 JH4022 | CACTGCTGGTTTGGAGCTCCAT |
| JH4023 | TGGGACAGTCTCTGGATGGCCT |
| F4 JH6159 | TCCAGAGACTGTCCCACCTTG |
| JH6160 | CGAGGACAGCCATACTAGGCT |
| F5 JH4082 | CTCGGAGAGGCTCTGCCAGCAT |
| JH4083 | AGCTGTCAGAACATGCATGTC |
| F6 JH6161 | GACATGCATGTTCTGACAGCT |
| JH6162 | CAGCTGCCTGGCTCAACAG |
| F7 JH6163 | TGAGCCAGGCAGCTGCCCTGT |
| JH6164 | GTGACACACTTCCTCCAGCT |
| F8 JH6165 | CTCATTTGGTGTGGCCAAGCT |
| JH6166 | ACTTGGCTTGGCTTGGCTTGAG |
| F9 JH6167 | CTGCTGCCTGTCAATCCAAATG |
| JH6168 | TGGGCTGGCTTCACATTTCAGA |

***DNA methylation***

| | |
|---|---|
| *Platr10 promoter* | |
| SJ1131 | TAATGATTTTTGTATATGTTATAGGTG |
| SJ1132 | TAATCTTACTTCTTTTACATATCTATA |
| SJ1133 | TGGGAGTTTGTTTAGTTATTTTGGAGT |
| SJ1134 | TTTGAGTAGATTAGTTTGTTTTGGTTT |
| | |
| *Oct4 promoter* | |
| SJ629 | GGATAGGTCGAGAGGGTGTAGTGTT |
| SJ630 | CACCCTCTAACCTTAACCTCTAAC |
| SJ631 | GTGGGATTGGGGAGGGAGAGGTGAA |
| SJ632 | TCCAAACCCACCTAAAAACCCTTAA |
| SJ633 | TTTGTTTGTTAAAGGTGGTGATGGT |
| SJ634 | ATAACCTACATAACAAAAACCAAAC |

***Platr10 CRISPR Cas9 knockout***

| | | |
|---|---|---|
| *Platr10* SpCas9 gRNA | | |
| gRNA1 | GCTCTCAGCTGTATCAGAGT | |
| gRNA2 | GGTGAACTCTAACCTGGAGT | |
| *Platr10* NmCas9 gRNA | | |
| gRNA1 | TGTCAGTAGAGCCTAGAAGAAAGT | |
| gRNA2 | CCTGATAGTTCGGACCCATTGTAT | |
| Cas9 control gRNA | | |
| shCT | GAAGTGGGATGATCCTCTGA | |
| | | |
| *Platr10* arm primers | | |
| ARM1-F (SJ2150) | AGTGCATAACTGGTTAATACTTGCT | |
| ARM1-R (SJ2152) | CAGCCCGTGAAATAATGCTGCA | |
| ARM2-F (SJ2153) | CCATCTCTTTATTCCCAAGGGTTC | |
| ARM2-R (SJ2154) | CTCAGGAGGAACACAGGTACTGTC | |
| | | |
| Recombination PCR/sequencing | | |
| SJ2155 | CTGAGAAATTTCAAGCTGTCTGC | 888bp |
| SJ1667 | CCATAAGGTCATGTACTGGGCA | |

## SUPPLEMENTARY FIGURE LEGENDS

**Figure S1. Mapping of the *Oct4* interacting lncRNAs by chromatin-RNA *in situ* reverse transcription trap sequencing (CRIST-Seq).**

A. CRIST-seq assay. The CRIST assay combines the specificity of Cas9 gene targeting with the simplicity of biotin-lncRNA labeling. Cells carrying the CRISPR Cas9 Oct4-gRNA cassette were first treated with formaldehyde to fix the Cas9 gRNA-Oct4 promoter chromatin complex structure. The promoter-interacting RNAs were *in situ* reverse transcribed into biotin-cDNAs (cDNAs) in the isolated nuclei with biotin-dCTP. The promoter chromatin-lncDNA complex was then isolated by Cas9 immunoprecipitation and was subsequently purified form genomic DNA by biotin-streptavidin bead purification. The CRIST-captured cDNAs were used for library construction. Illumina sequencing was used to identify lncRNAs that interact with the *Oct4* promoter.
B. CRIST targeting and control vectors. pEF1: EF1-alpha promoter; dCas9: the catalytically inactive CRISPR Cas9; gRNA1-2: Cas9 guiding RNAs that target the *Oct4* promoter; U6: RNA polymerase III U6 promoter; H1: human H1 RNA polymerase III promoter; gCT: a random gRNA control. Two Cas9 gRNAs are transcribed by U6 and H1 promoters, respectively, and guides Cas9 to the *Oct4* promoter. Cas9 gCT and empty vector were used as the CRIST controls.
C. The targeting and control sites in the *Oct4* promoter. pOct4: Oct4 promoter where the Cas9 gRNAs are designed; 5'-Ct: the 5'- 3C negative control site that is 13.9 kb away from the Cas9 gRNA targeting site.

D. Off-target control. A 5’-upstream site of the GAPDH house keeping gene was used as the off-target control site.
E. Specificity of the CRIST targeting. The CRIST signals were detected by qPCR at the 5’-Ct, pOct4 targeting site and the off-target site. Vector: cells that were treated with the Cas9 control vector that lacks the gRNAs; gRNA: cells that were targeted by both Cas9 and *Oct4* gRNAs; gCT: cells that were treated with the random control gRNA vector. Throughout the manuscript, the data are presented as the mean ± SD from three independent experiments unless they are specifically defined. ** $p<0.01$ as compared with Vector and gCT controls.

**Figure S2. Location of the Cas9-Oct4 gRNAs in the *Oct4* promoter.**

A. Diagram of the CRISPR Cas9-guided chromatin immunoprecipitation (Cas9-IP) vector. Cas9: CRISPR Cas9; gRNA: Cas9 guiding RNAs that target the *Oct4* promoter (sequences under the diagram); pEF1: the human EF-1a promoter; pH1: human H1 promoter; pU6: U6 promoter; T5: the TTTTT termination signal of RNA polymerase III.
B. Location of the two *Oct4* promoter Cas9 gRNAs. *Oct4* mRNA is shown in green and the coding sequences in red. Two Cas9 gRNAs are highlighted in yellow and PAM sequences in red in the sequence of the *Oct4* promoter region. gRNA1 is located immediately the upstream of the TF binding site.

**Figure S3. CRIST targeting of the *Sox2* promoter.**

**A.** Location of the two Cas9 gRNAs in the *Sox2* promoter. Two *Sox2* Cas9 gRNAs are highlighted in yellow and PAM sequences in red in front of the *Sox2* transcription initiation site (TS+1).

B. CRISPR Cas9 *Sox2*-gRNA vector and control vectors. dCas9: defective CRISPR Cas9; gRNA1, 2: two Cas9 guiding RNAs that target the *Sox2* promoter.

C. Specific targeting of the *Sox2* promoter. pSox2: the Cas9 gRNA targeting site in the *Sox2* promoter; 5'-Ct: a control site that is 14.6 kb away from the pSox2 target site; Off-target: a CRIST control site that is 33.8 kb upstream of the housekeeping gene GAPDH. Cas9 enrichment signals were quantitated by qPCR using specific primers derived from the pSox2 targeting site, 5'-Ct control site and off-target site. All data shown are mean±SEM from three independent experiments after normalization over the IgG control. ** $p<0.01$ as compared with the Cas9 Vector and Cas9-gCT controls.

**Figure S4. Gene structure of *Platr10*.**

A. The IGV plot of *Platr10* from RNA-seq data of iPSCs and fibroblasts. *Paltr10* (Gene ID ENSMUSG00000099370) is located at chromosome 3: (chr3:75,647,445-75,655,731). Both the iPSC and fibroblast RNA-seq Bam data were analyzed by IGV, and the Sashimi plot was acquired for *Platr10*. Note differential expression of *Platr10* in two cell populations.

B. The location and exons of *Platr10*.

**Figure S5. Characterization of *Platr10* variants.**

A. The 5'- and 3'-RACE of *Platr10* lncRNA. After racing, the PCR bands were cut and cloned in pJet vector for sequencing.
B. Diagram of the two alternatively spliced variants.
C. The sequences of NONMMUT043505 variant 1 (V1). The underlying capital letters in red are the sites of intron splicing. V1 contains an extra exon as compared with variant 2 (V2).
D. The sequences of NONMMUT043505 variant 2 (V2).

**Figure S6. Expression abundance of *Platr10* and promoter DNA demethylation in reprogramming.**

A. The CpG islands in the *Platr10* promoter. E1-E4: exons 1-4 of the *Platr10* gene. Two CpG islands in the *Platr10* promoter were selected for the measurement of DNA methylation
B. Sodium bisulfite sequencing. Cells were collected during the process of reprogramming. Genomic DNA was isolated and treated with sodium bisulfite to convert the unmethylated cytosines into uracils, while the methylated cytosines were not affected. The treated DNA was amplified, cloned into pJet vector, and sequenced. Open circle: unmethylated CpG; solid circle: methylated CpG. The *Platr10* promoter DNA becomes demethylated in iPSCs, which is associated with the activation of *Platr10* in reprogramming.

**Figure S7. Functional studies of *Platr10* lncRNA.**

A. *Platr10* shRNA knockdown vectors. pH1: human H1 promoter; pU6: U6 promoter; T5: the TTTTT termination signal of RNA polymerase III. Two lentiviral shRNA vectors (shPlatr10-1 and shPlatr10-2) are constructed to knockdown *Platr10*. Each lentiviral vector carries two shRNAs under the control of pH1 and pU6 promoters to target *Platr10* lncRNA.

B. Knockdown of *Platr10* by shRNA in E14 cells. shPlatr10-1, -2: shRNA lentiviruses. shCT: random shRNA control; Vector: lentiviral empty vector control. The abundance of *Platr10* was measured by qPCR and the value of the vector control was set as 1. ** $p < 0.01$ as compared with the Vector and shCT controls.

C. *Platr10* enhances cell reprogramming. MEF cells were transfected with the lentiviruses carrying the empty vector (Vector), lncRNA control (CTL), and *Platr10*. Doxycycline (DOX) was added in the medium to initiate reprogramming. iPSC colonies were immunostained using anti NANOG antibody (green).

**Figure S8. Genomic deletion of *Platr10* by CRISPR Cas9 targeting.**

A. Schematic diagram of *Platr10* targeting. SpCas9: Streptococcus pyogenes Cas9; NmCas9: Neisseria meningitides Cas9; gRNA: Cas9 guiding RNA; pCMV: CMV promoter; pU6: human U6 promoter; pH1: RNA polymerase III H1 promoter; GPF: copGFP; Puro: puromycin; Arm1, Arm2: two *Platr10* genomic sequences used for Cas9-mediated recombination. Under the guidance of gRNA, Cas9 mediated genomic recombination at the *Platr10* locus, resulting in the

deletion of the region covering the *Platr10* promoter and coding region. Cells were treated with puromycin for positive selection and ganciclovir for negative selection. The copGFP-positive cells were selected and validated by PCR DNA sequencing.

B. Cas9-targeted E14 cells. *Platr10*-deleted cells were copGFP-positive (red arrow). Cell morphology was altered in the *Platr10*-gRNA group, but not in the gRNA control group (while arrow).
C. Loss of the pluripotent marker NANOG in *Platr10*-deleted cells. Expression of the pluripotent maker NANOG was detected by immunostaining. Note the loss of the pluripotent NANOG marker in *Platr10*-deleted cells (red arrow), but not in control cells (white arrow).

**Figure S9. Characterization of *Platr10* Oct4 binding element.**

A. The sequence of Oct4 binding element (OBE) of *Platr10* lncRNA. In the IGV program, each sequencing read from the peak region was downloaded and a 50 bp consensus sequence was taken for the OBE of *Platr10*.
B. The predicted structure of the *Platr10* OBE. The structure of OBE was obtained by submitting the OBE sequence to the RNA structure prediction website:https://rna.urmc.rochester.edu/RNAstructureWeb/Servers/Predict1/ResultsPages/20180916.191929-72078a8d/Results.html.

**Figure S10. Nuclear localization of *Platr10*.**

A. Subcellular localization of *Platr10* by RT-PCR. Cytoplasmic and nuclear RNAs were isolated and used for RT-PCR of cellular *Platr10* on agarose gel. Nuclear

U2 was used as the positive control.

B. Quantitation of subcellular *Platr10* by qPCR. β-Actin was used as the cytoplasmic control and U6 was used as the nuclear control.

C. Chromatin-bound *Platr10*. Chromatin and nucleoplasm fractions were isolated and RNAs in each fraction were purified for qPCR. Note the primary location of *Platr10* in the chromatin fraction.

D. *Platr10* RNA FISH. *Platr10* lncRNA in E14 cells was detected with Ribo lncRNA FISH Probe Mix (red, Guangzhou RiboBio, China). The nuclear DNA was stained with DAPI (blue). shCT: E14 cells that were transfected with shRNA random control; shPaltr10-1: cells that were treated with shPlatr10-1 shRNA.

**Figure S11. Specific interaction of *Platr10* with multiple stemness genes.**

The RAT-seq bam data were converted to coverage and uploaded to the UCSC browser. The IGV plot shows the specific binding of *Platr10* lncRNA to *Oct4* (A), *Sox2* (B), *Klf4* (C), and *c-Myc* (D). RAT-CT: RAT-seq control, in which the RAT library was constructed using two random oligonucleotides, instead of specific antisense oligonucleotides from *Platr10*. *Palr35*: the RAT-seq lncRNA control. LncRNA *Palr35* is also differentially expressed in correlation with reprogramming. Like *Platr10*, it is silenced in fibroblasts and becomes activated in iPSCs. However, unlike *Platr10*, it does not bind to the stemness gene loci (top panel). These data suggest that the binding of *Platr10* to the stemness gene loci is lncRNA-specific.

**Figure S12. Confirmation of the *Platr10-Oct4* interaction by RAT and ChIRP assays.**

A. *Platr10-Oct4* interaction by RAT-qPCR. The RAT samples were used to quantitate the binding intensity of *Platr10* lncRNA to the *Oct4* promoter. The results were normalized to the value of the RAT control that was constructed using random oligo primers. 5'-CT: the 5'-upstream control site; pOct4: Oct4 promoter; shPlatr10-1, 2: two shRNAs used to knockdown *Platr10*; shCT: random shRNA control; *Palr35*: control RAT using primers from lncRNA *Palr35* that is differentially expressed in reprogramming, but it is not in the *Oct4* CRIST-seq list. ** $p < 0.01$ as compared with shRNA-treated groups and control lncRNA group.
B. Validation of the *Platr10-Oct4* interaction by the ChIRP assay (Chromatin Isolation by RNA Purification). After cell lysis and sonication, the *Platr10* chromatin complex was hybridized to biotinylated oligonucleotide probes and was pulled down with streptavidin beads for quantitation of the *Platr10*-interacting target signal. Random: the ChIRP control using random biotinylated oligonucleotide DNA probes; *Platr10*: *Platr10* biotinylated oligonucleotide DNA probes. pOct4: the *Oct4* promoter; 5'-CT: the 5' upstream control region of *Oct4*. For comparison, the Random control group was set as 1. ** $p<0.01$ as compared with the 5'-CT site.

**Figure S13. Confirmation of the *Oct4* 3C loop products by DNA sequencing.**

The *Oct4* intrachromosomal loop was examined by 3C PCR. The 3C products were sequenced. Blue line on the top of the sequence: the 3C ligation product between the

ligated BamH1 and Bgl2 sites.

**Figure S14. Confirmation of the TET1-*Platr10* interaction by CLIP and RNA pulldown MS sequencing.**

A. *Platr10* RNA pulldown MS sequencing. The biotin-labeled *Platr10* sense lncRNA was used to pull down its binding proteins in E14 cells. The *Platr10* antisense RNA was used as the control. MS fragment sequences were analyzed using PEAKS 7 with a false discovery rate (FDR) threshold <5%. *Platr10*-bindnig proteins were calculated as the protein enrichment ratio (the PEAKS score, −10logP) after adjusting over that of the antisense control.

B. The TET1 and *Platr10* interaction by CLIP. E14 cells were UV cross-linked in a CL-1000 UVP UV cross-linker oven and were lysed with the lysis buffer. After sonication, immunoprecipitation was performed with anti-TET1 antibody. The precipitated RNAs were used for qPCR to determine the TET1-*Platr10* interaction. IgG: the CLIP control. For comparison, the IgG group was set as 1. ** $p<0.01$ as compared with control groups.

**Figure S15. Confirmation of the TET1-*Oct4* interaction by ChIP.**

A. Location of ChIP primers. pOct4: the *Oct4* promoter; 5'-CT: the 5'- control region.

B. TET1-Oct4 interaction by ChIP. The ChIP signal was adjusted over the IgG group and set the 5'-CT as 1. ** $p < 0.01$ as compared with shPlatr10-treated groups.

**Figure S16. Mutation of *Platr10* OBE and TBE elements.**

A. Sequences and locations of OBE and TBE in *Platr10* lncRNA. E1-E4; *Platr10* exons; OBE50: 50 bp Oct4 binding element; TBE58: consensus TET1 binding element.

B. *Platr10* mutation constructs. F947: the full length *Platr10* lncRNA; OBE50D: *Platr10* lncRNA that lacks the 50 bp Oct4 binding element; TBE58D: *Platr10* lncRNA that lacks the 58 bp TET1 binding element; TBE58M: *Platr10* lncRNA that has the replacement of 58 bp TET1 binding element with a random 58 bp DNA sequence.

C. *Oct4* promoter-luciferase assay. The *Oct4* 3.9K promoter-luciferase vector was co-transfected into 293T cells with the vectors that carry the full length *Platr10* (1041bp), OBE50D that lacks the 50 bp *Oct4* binding element (OBE), TBE58D that lacks the 58 bp consensus TET1 binding element (TBE), and TBE58M that contains a mutant TBE. Forty-eight hours after co-transfection, cells were collected for the measurement of luciferase activity. The data are the mean ± SD from three independent experiments. * $p < 0.01$ as compared with the Vector control; ** $p < 0.01$ as compared with the full length *Platr10*. Deletion of the 50 bp *Oct4* binding element abolishes the function of *Platr10* in the activation of the *Oct4* promoter-luciferase reporter vector.

**Figure S17. *Platr10* partially corrects LIF withdrawal-induced defects in E14 cells.**

A. Diagram of the LIF-withdrawal model. E14 cells were treated three times with lentiviruses carrying *Platr10* and its mutants. After puromycin selection, cells

were reseeded at very low density in DMEM medium that lacks LIF. Stem cell spheroid formation and expression of pluripotent marker *Nanog* were measured.

B. Formation of stem cell spheroids. Images were taken 5 days after LIF withdrawal. After LIF withdrawal, E14 cells at low density failed to thrive to form stem cell spheres. However, the wild type *Platr10* but not its mutants was able to partially correct this defect.

C. Expression of pluripotent marker gene *Nanog*. Cells were collected for qPCR quantitation of *Nanog*. The wild type *Platr10* reduced the loss of *Nanog* induced by LIF withdrawal. ** $p < 0.01$ as compared with LIF- and mutant groups.

**Figure S18. *Platr10* regulates DNA demethylation in the *Oct4* promoter**

A. CpG islands in the *Oct4* promoter. 5'-Enh: the 5'-enhancer; pOct4: *Oct4* promoter; E1-E5: *Oct4* exons; CpGs: CG dinucleotides.

B. Knockdown of *Platr10* induces CpG demethylation in the *Oct4* promoter. shCT: random shRNA control; Vector: empty vector with shRNA; shPlatr10-1, -2: shRNAs targeting *Platr10*. Solid circle: methylated CpG. After shRNA knockdown, E14 cells were collected and genomic DNAs were extracted and treated by sodium bisulfite. The *Oct4* promoter DNAs were amplified with *Oct4*-specific primers and cloned into the pJet vector for sequencing. Each line represents sequencing from one clone.

C. *Platr10* partially corrects the altered epigenotype induced by LIF-withdrawal. E14 cells were transfected with *Platr10* and cultured at very low density in the $LIF^-$ medium to induce differentiation. Sodium bisulfite sequencing was used to compare the status of DNA methylation in the *Oct4* promoter. After LIF withdrawal, the *Oct4* promoter became methylated. Overexpression of *Platr10*,

but not its mutants, partially corrected the LIF withdrawal-induced defect. F1041: full length *Platr10*; D58: the 3'-end deleted *Platr10*; M58: the 3'-end mutated *Platr10*; Vector: lentiviral expression vector control.

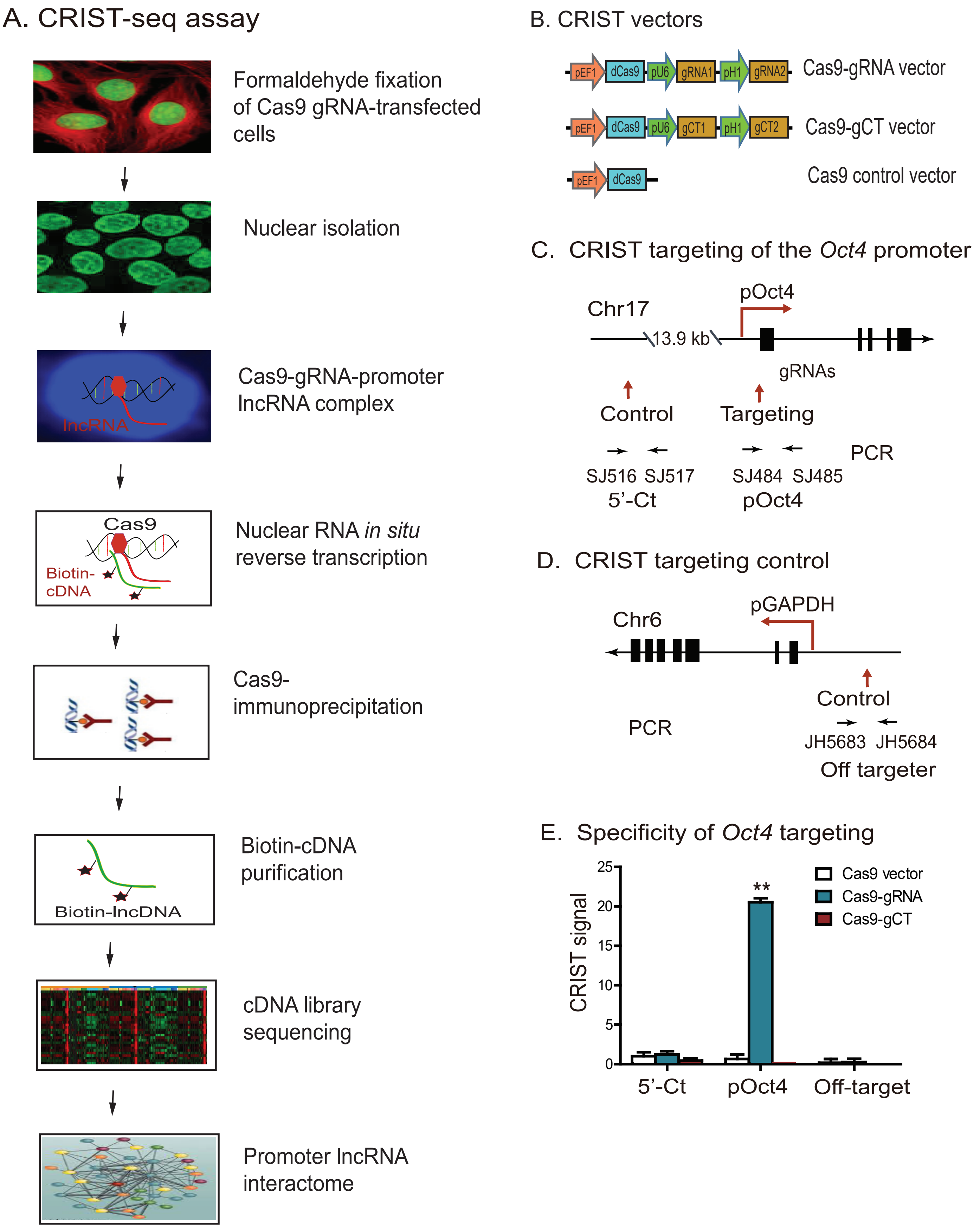


Figure S1. CRIST-Seq to map the *Oct4* promoter RNA interactome

A. CRIST-seq vector

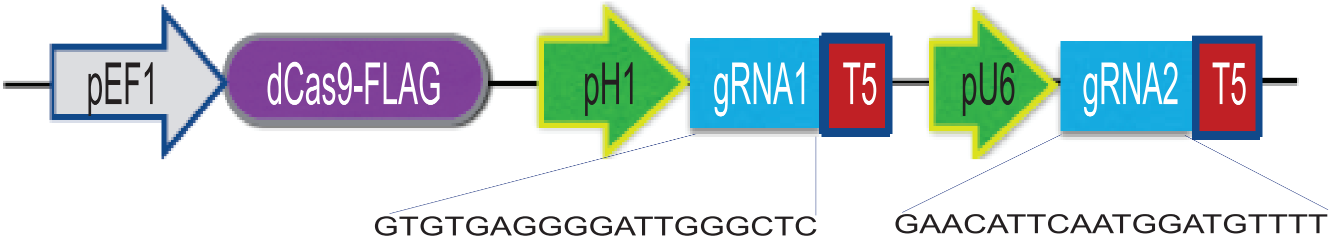


B. Location of CRIST Cas9 gRNAs

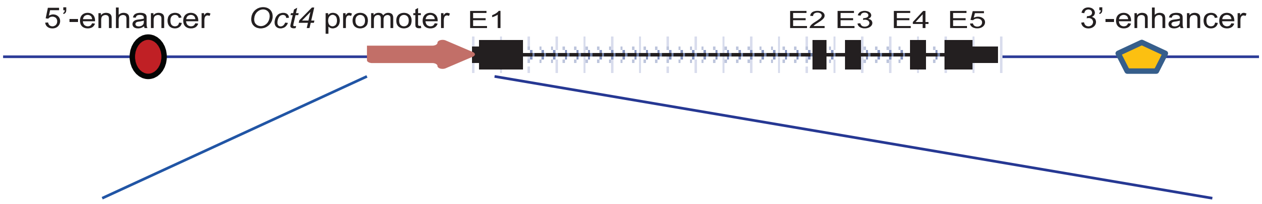


```
42961 aggatgaaca ccggagtccc tggaggaagg gaagcagggt atctccatct gaggctctgt
43021 ctttgaggag aggtggagag ctggggaagt cttGTGTGAG GGGATTGGGG CTCAGGaggg   gRNA1-PAM
43081 ggttggggag caggaagttg tccccagggg agccatcctg gcccattcaa gggttgagta   TF binding
43141 cttgtttagg gttagagctg ccccctctgg ggaccaggat tgtccagcca aggccattgt   site
43201 cctgccccct tcccccagtc cctcccaggc ccctttgaac ctgaagtcag atatttcttc
43261 tctctaccca cctcccaccc gttgggtttc tccacccagg aactaggctg gaagcctggg
43321 atgaggaggt gggggaggg agaactgaga atcttgagga aagaggcccc ggccttaact
43381 gtgaggggat ggagcctggg tgcaggtctt atggggttg ggggtggtta gtgtctaatc
43441 taccaacctg gacaacacaa gatggaatac tgtgctctga aaacgcagag ccagcacttc
43501 tctgggatct ctggggacat atctggttgg ggctcgggt cccatggtgt agagcctcta
43561 aactctggag gactggaggt gcaatggctg tcttgtcctg gccttggaca tgggctgaaa
43621 tactgggttc acccatatct agaactctag acgggtgggt aagcaagaac tgaggagtgg
43681 ccccagaaat aattggcaca tGAACATTCA ATGGATGTTT TAGGctctcc agaggatggc   gRNA2-PAM
43741 tgagtgggct gtaaggacag gccgagaggg tgcagtgcca agaggccttg tggtgcgatg
43801 gggcatccga gcaactggtt tgtgaggtgt ccggtgaccc aaggcagggg tgagaggacc
43861 ttgagggttg aaaatgaagg cctcctgggg tcccgtccta agggttgtcc tgtccagacg
43921 tccccaacct ccgtctggaa gacacaggca gatagcgctc gcctcagttt ctcccacccc
43981 cacagctctg ctcctccacc cacccagggg gcgggggccag aggtcaaggc tagagggtgg
44041 gattggggag ggagaggtga AACCGTCCCT AGGTGAGCCG TCTTTCCACC AGGCCCCCGG   TS +1
44101 CTCGGGGTGC CCACCTTCCC CATGGCTGGA CACCTGGCTT CAGACTTCGC CTTCTCACCC
```

*Oct4* coding region

Figure S2. Location and sequnce of *Oct4* Cas9 gRNAs

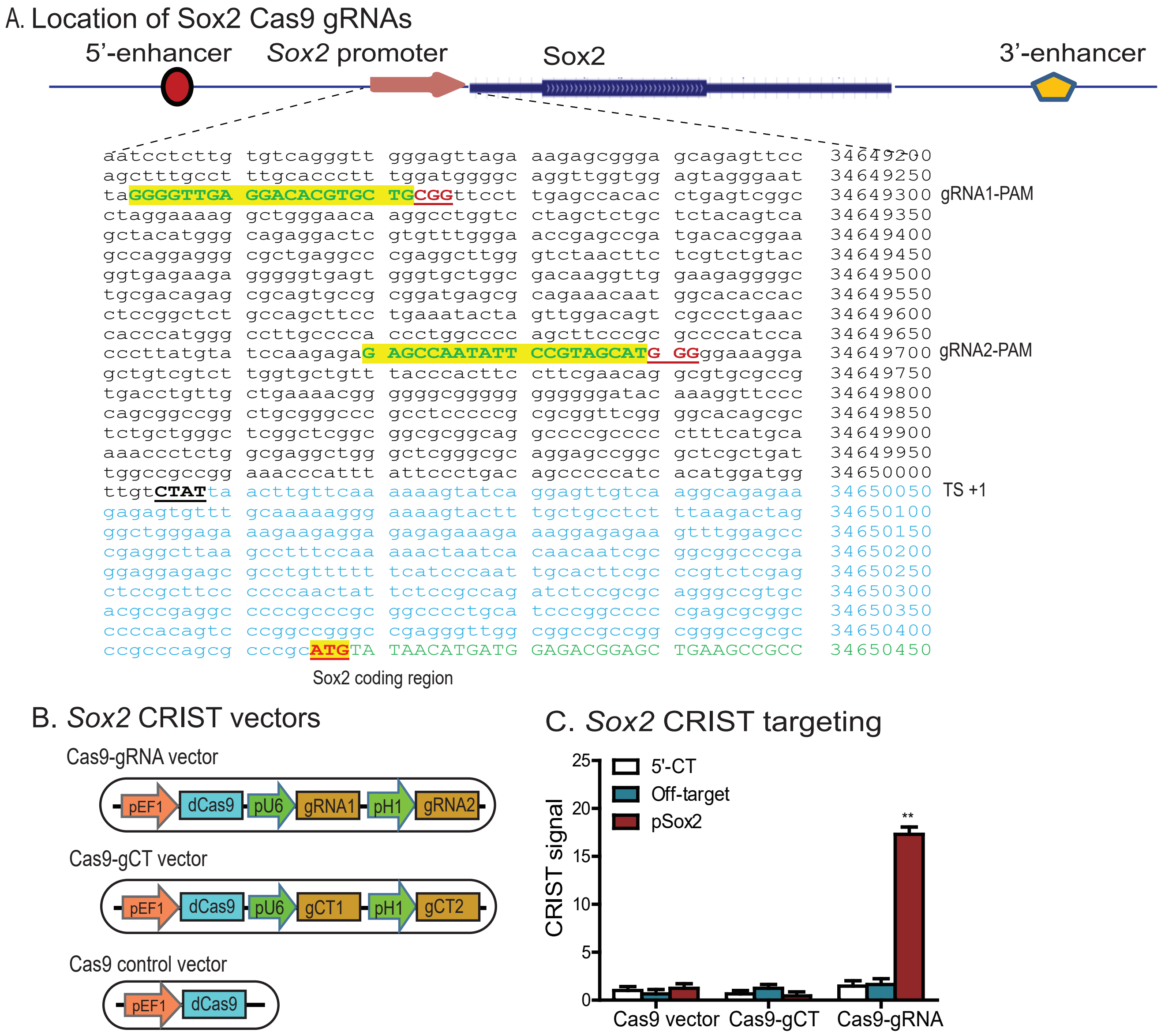


Figure S3. *Sox2* CRIST-seq targeting

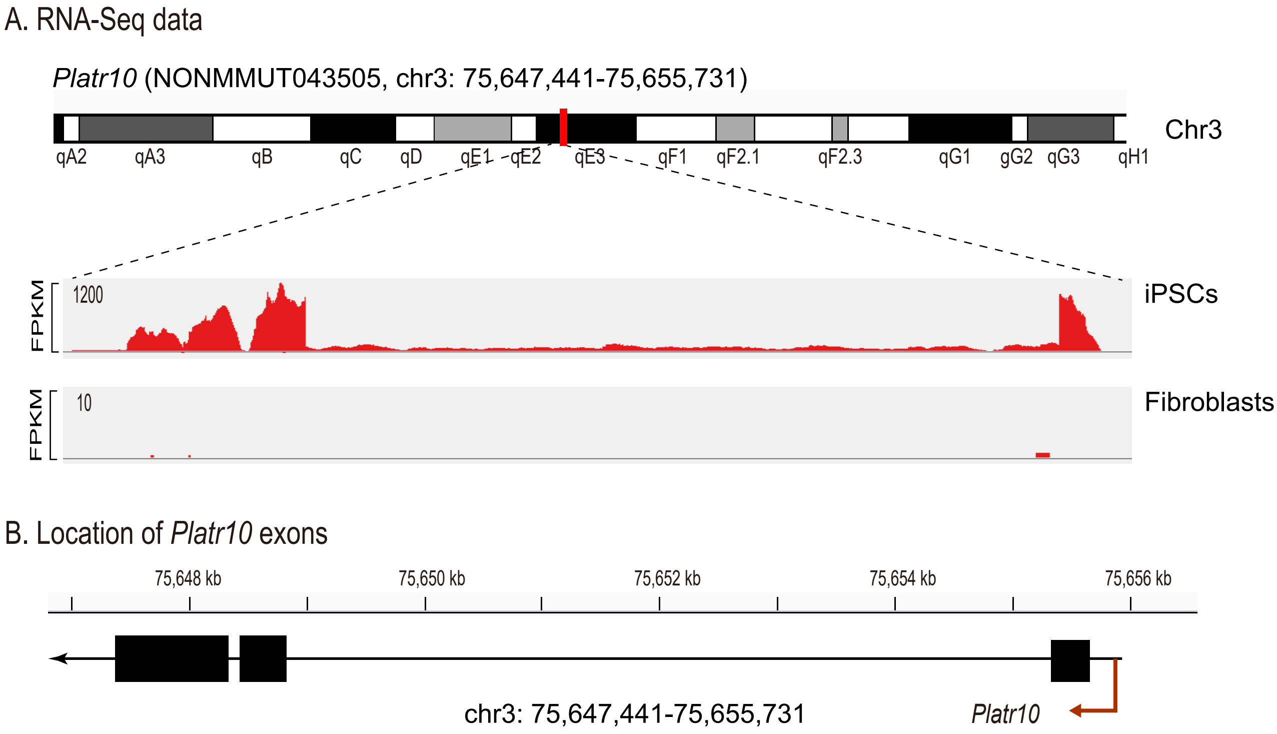


Figure S4. *Platr10* RNA-seq IGV data

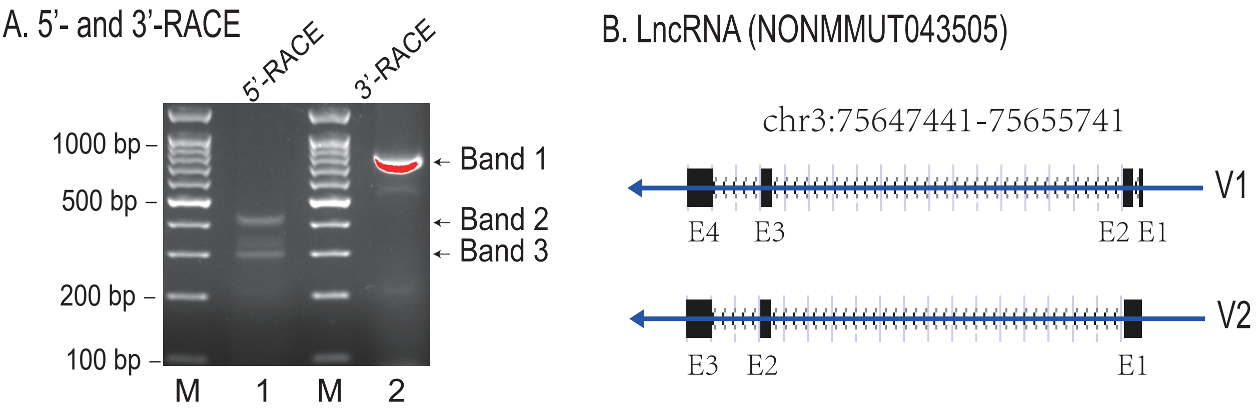


## C. NONMMUT043505 variant 1

tagctattctggagcctacacgtggtcacctgaagctggctccaaggatgatttggtgggaatgggcttcccctctc
ctttataaaTtccgagatgcctcccaggctcgaacccggacatgagagccgcaggccggccccaaccatttggcgaa
ccaacttgggacttaggaatggcagaggacatttggaagaaacactgctggtttggagctccatggaagaagaaaag
aattaaagaaaattcataccggagaaGgaaacacagagacaaaaaatggagcagactgaaggaaaggccatccagag
actgtcccaccttgtgattcttcccatctgcagacaccaaaccctgacactattgctgataccaagacgtactccca
gacaggagcctagtatggctgtcctcggagaggctctgccagcatctgactaagatagatgcaGctcctggattcta
tgatgttcttttagaagtatgtagctgcctcttgtggaagatagacatgcatgttctgacagctcaaagaaaaaatt
atttatggagaattgttgccaaacagcctgctgtggctcttagagcatttctgtgtatctgttgagccaggcagctg
ccctgtcatgcagactgaatgtttaagtgcattgtggttggctttggtggtttgtatgaaaatagcctttgtaggct
catttggtgtggccaagctggaggaagtgtgtcactactgggggtgggctttgaggtttcaaatgctcaagccaag
ccaagccaagtgtccctctttcttcctgctgcctgtcaatccaaatgtagaagtcttagctacctctccagcatcat
gtctgccttcatgctgccatgcttcctaccatggtaagaatggactaaacctctgaaatgtgaagccagcccaaata
aaatgttttcctttttaactgttaaaaaaaaaaaaaaa

## D. NONMMUT043505 variant 2

tagctattctggagcctacacgtggtcacctgaagctggctccaaggatgatttggtgggaatgggcttcccctctc
ctttataaagtgtgtctgccattaaaaatttgaaccttgagcagactagcttgtcttggttccattatttctctacc
tgcctagattccctcttctcttccagttccgagatgcctcccaggctcgaacccggacatgagagccgcaggccggc
cccaaccatttggcgaaccaacttgggacttaggaatggcagaggacatttggaagaaacactgctggtttggagct
ccatggaagaagaaaagaattaaagaaaattcataccggagaaGgaaacacagagacaaaaaatggagcagactgaa
ggaaaggccatccagagactgtcccaccttgtgattcttcccatctgcagacaccaaaccctgacactattgctgat
accaagacgtactcccagacaggagcctagtatggctgtcctcggagaggctctgccagcatctgactaagatagat
gcaGctcctggattctatgatgttcttttagaagtatgtagctgcctcttgtggaagatagacatgcatgttctgac
agctcaaagaaaaaattatttatggagaattgttgccaaacagcctgctgtggctcttagagcatttctgtgtatct
gttgagccaggcagctgccctgtcatgcagactgaatgtttaagtgcattgtggttggctttggtggtttgtatgaa
aatagcctttgtaggctcatttggtgtggccaagctggaggaagtgtgtcactactgggggtgggctttgaggttt
caaatgctcaagccaagccaagccaagtgtccctctttcttcctgctgcctgtcaatccaaatgtagaagtcttagc
tacctctccagcatcatgtctgccttcatgctgccatgcttcctaccatggtaagaatggactaaacctctgaaatg
tgaagccagcccaaataaaatgttttcctttttaactgttaaaaaaaaaaaaaaaaaaaaaaaaaaaaa

Figure S5. Alternative splicing of lncRNA NONMMUT043505 (*Platr10*)

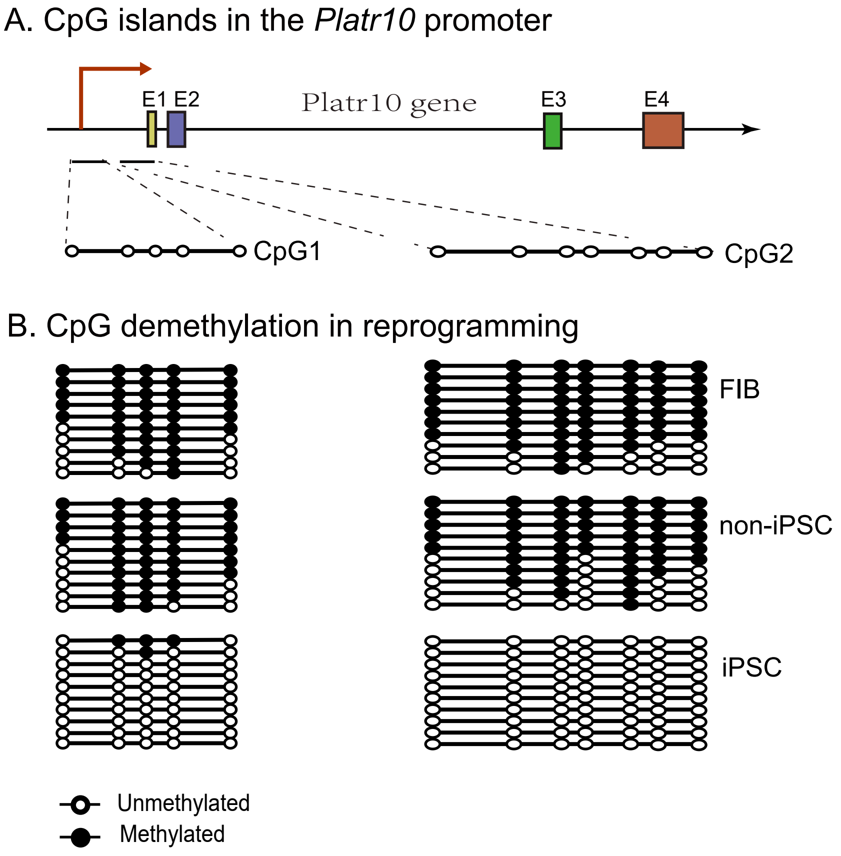


Figure S6. *Platr10* promoter DNA methylation

A. *Platr10* knockdown vectors

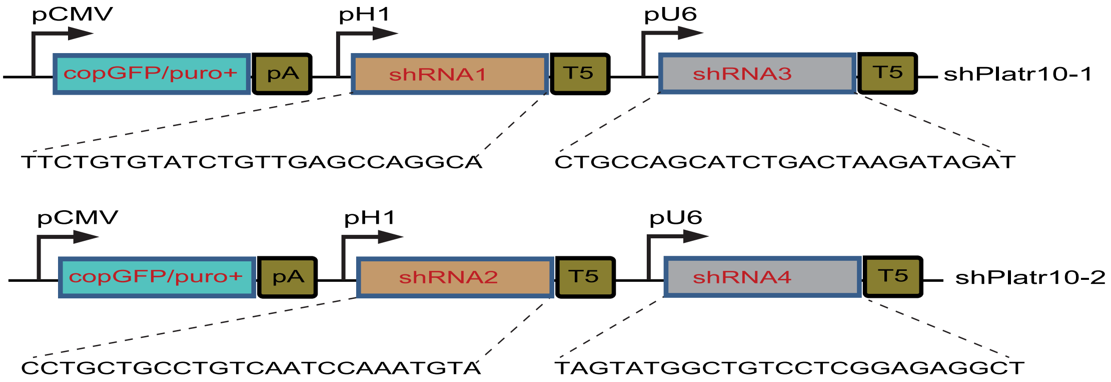


B. *Platr10* knockdown

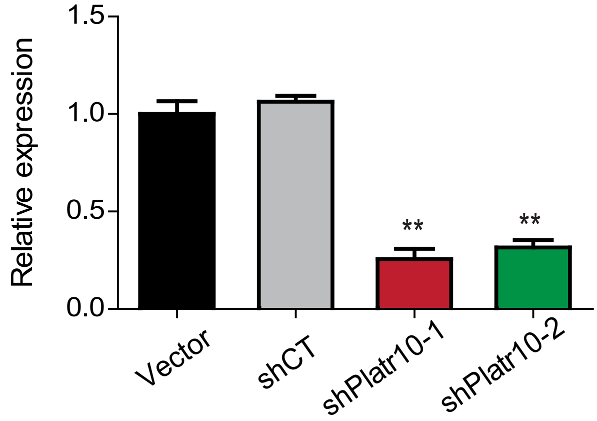


C. *Platr10* promotes reprogramming

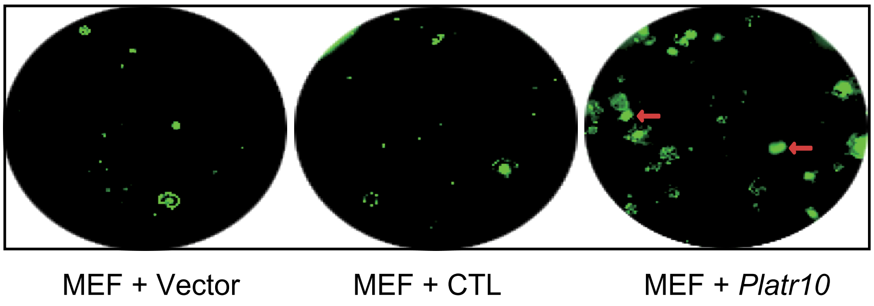


Figure S7. Functional studies of *Platr10*

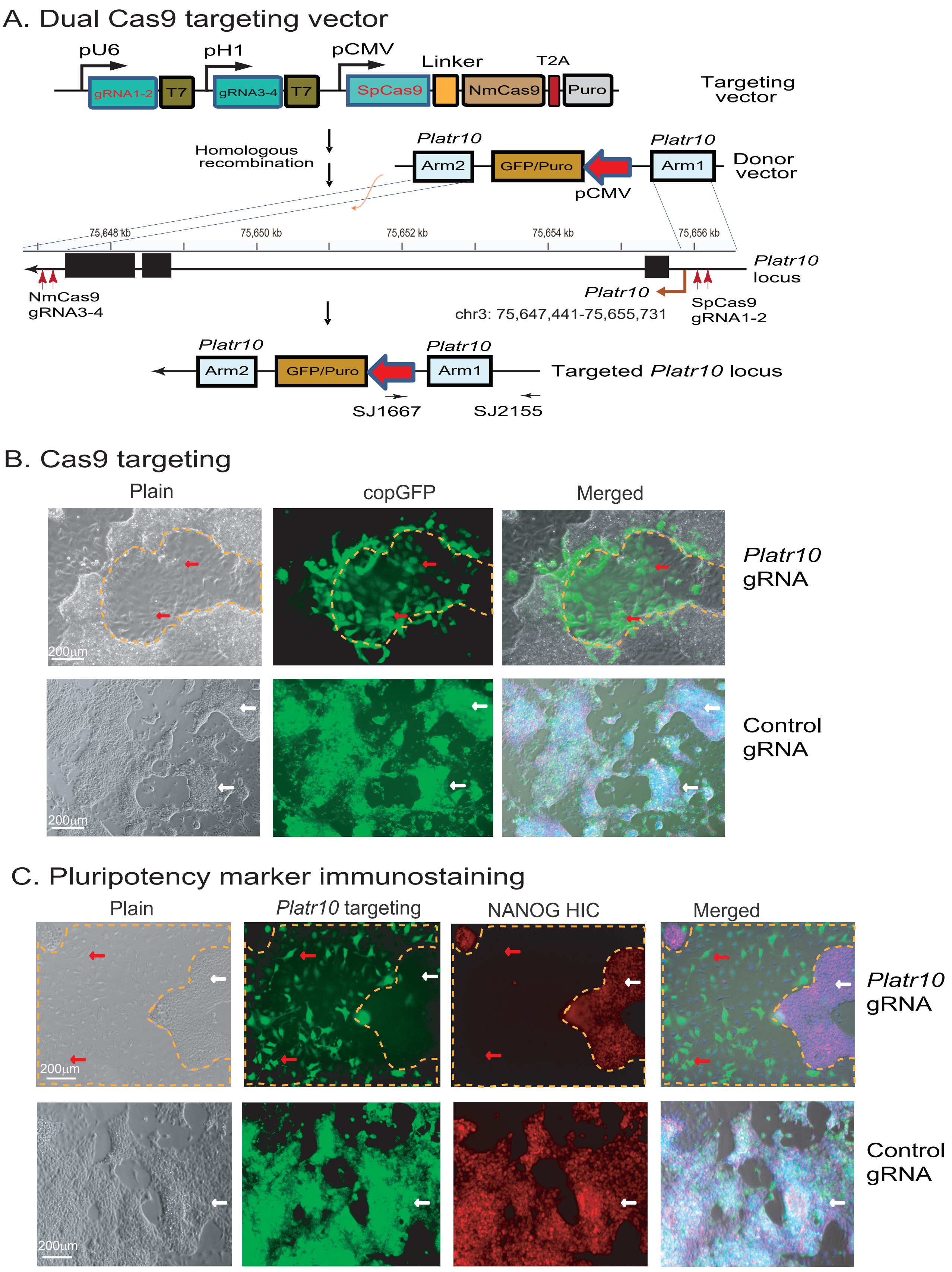


Figure S8. Dual Cas9 targeting of *Platr10* in E14 cells

A. *Platr10 Oct4* binding element

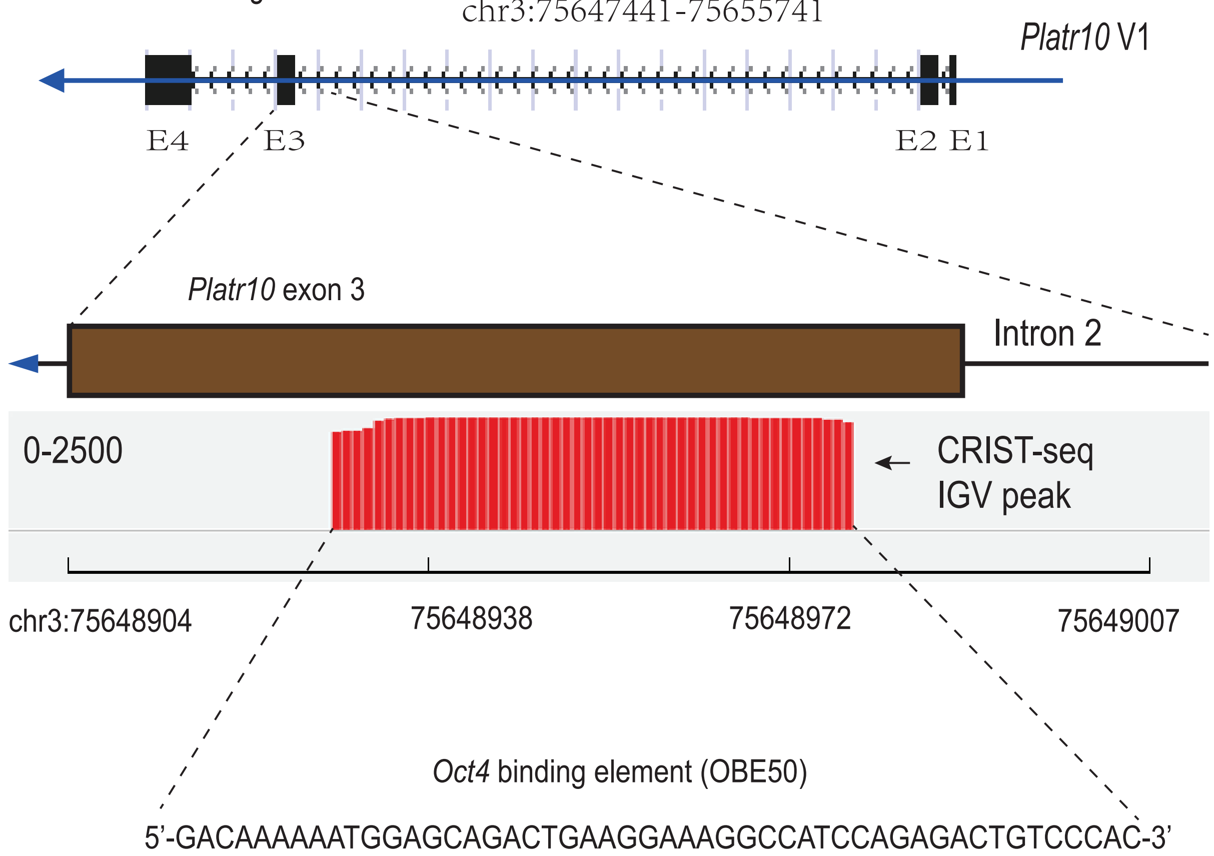


B. Predicted RNA structure of *Platr10* OBE50

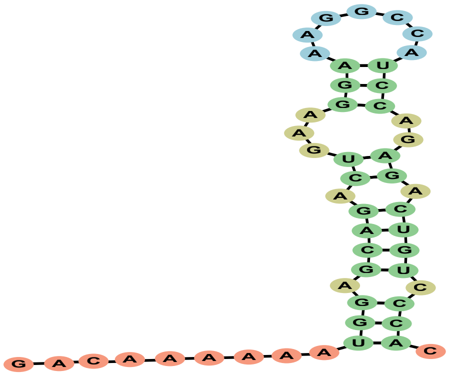

Figure S9. *Platr10 Oct4* binding element (OBE50) by CRIST-seq IGV

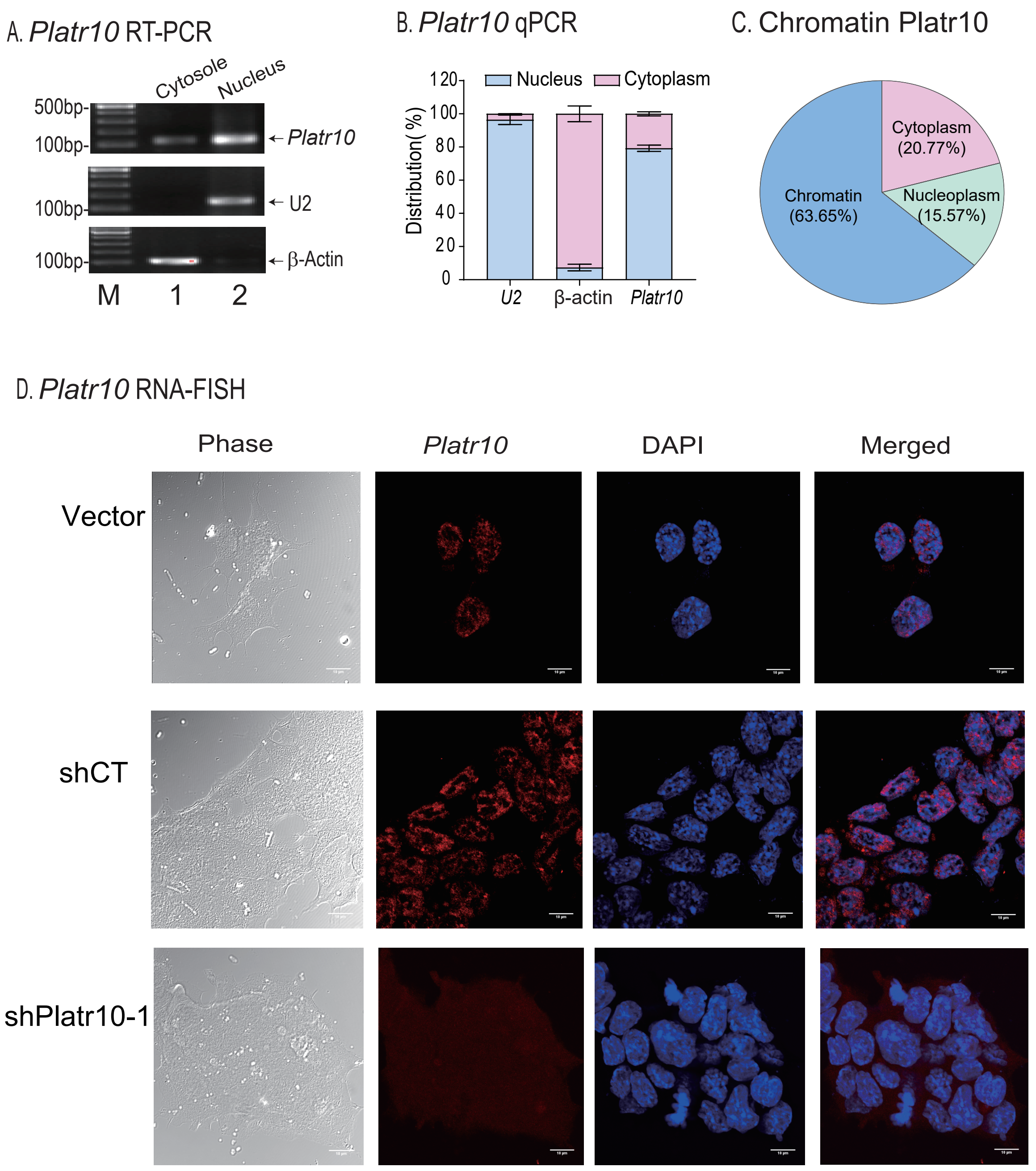


Figure S10. Nuclear localization of *Platr10*

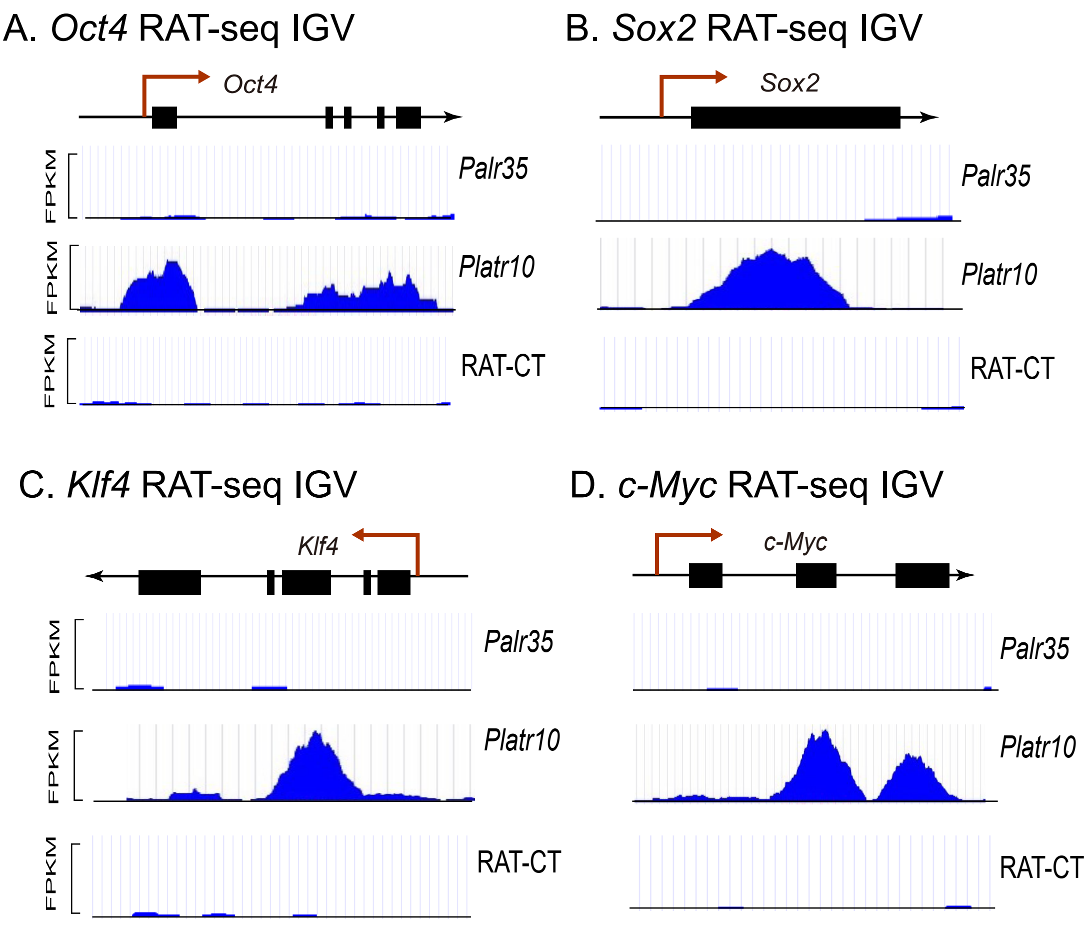


Figure S11. Specfiic binding of *Platr10* to stemness genes

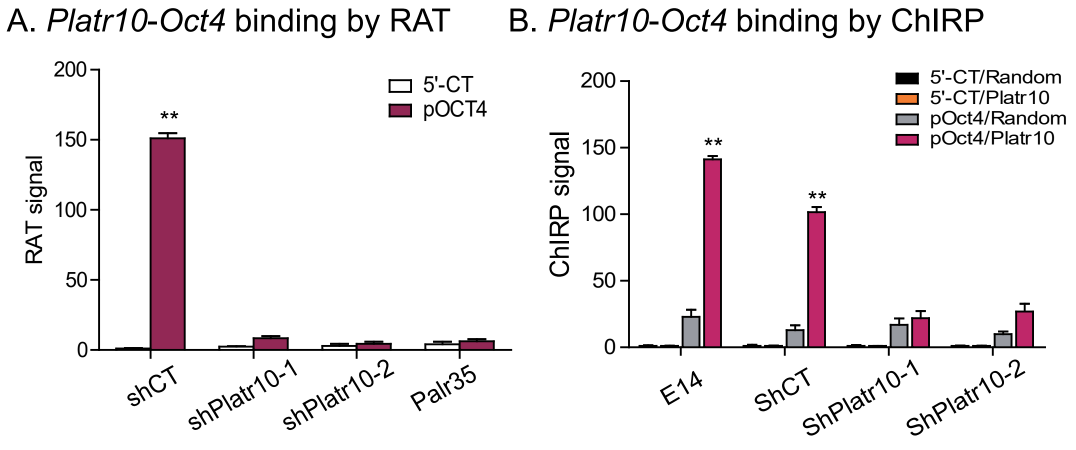


Figure S12. The *Platr10-Oct4* interaction

A. *Oct4* 3C sequencing

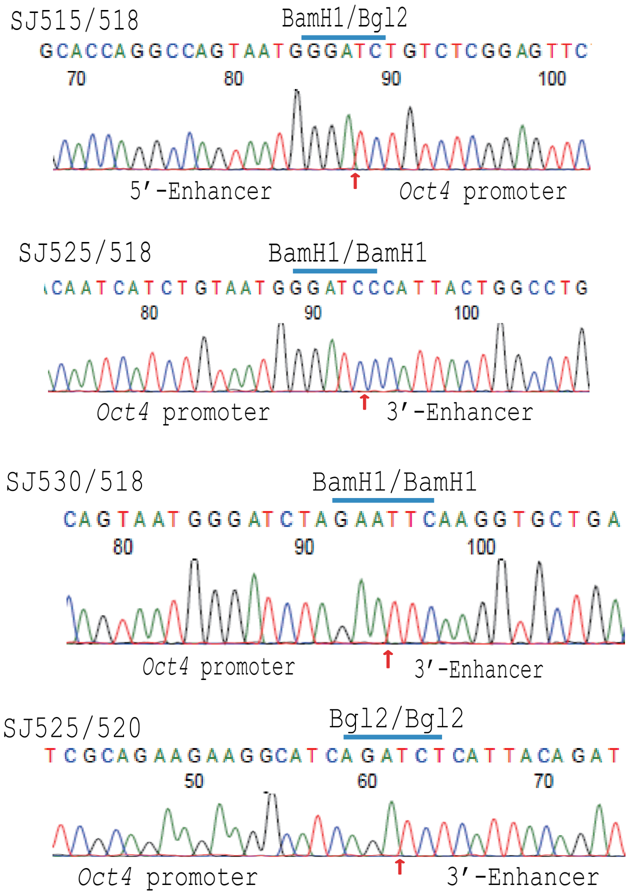


Figure S13. The 3C loop at the *Oct4* locus by DNA sequencing

## A. *Platr10* RNA pulldown-MS protein targets

| GO Biological pathway | Proteins | Gene name | -10lgP |
|---|---|---|---|
| DNA methylation and demethylation (5) | | | |
| | Methylcytosine dioxygenase TET1 | Tet1 | 220.6 |
| | Histone-lysine N-methyltransferase EZH2 | Ezh2 | 162.3 |
| | Transcriptional repressor CTCF | Ctcf | 170.0 |
| | Methylcytosine dioxygenase TET2 | Tet2 | 160.1 |
| | DNA (cytosine-5)-methyltransferase 1 | Dnmt1 | 231.5 |
| Stem cell population maintenance (3) | | | |
| | Catenin beta-1 | Ctnnb1 | 131.2 |
| | Transcription factor SOX-2 | Sox2 | 111.6 |
| | NF-kappa-B-activating protein | Nkap | 93.2 |
| Covalent chromatin modification (7) | | | |
| | Actin-like protein 6A | Actl6a | 122.9 |
| | Histone acetyltransferase KAT6B | Kat6b | 114.5 |
| | Bromodomain-containing protein 3 | Brd3 | 98.4 |
| | Histone acetyltransferase KAT7 | Kat7 | 92.1 |
| | Lysine-specific demethylase 3B | Kdm3b | 68.9 |
| | Histone chaperone ASF1A | Asf1a | 61.3 |
| | Histone acetyltransferase KAT8 | Kat8 | 34.8 |
| Chromatin remodeling and assembly (6) | | | |
| | Actin-like protein 6A | Actl6a | 122.9 |
| | AT-rich interactive domain-containing protein 1B | Arid1b | 96.6 |
| | Death domain-associated protein 6 | Daxx | 89.5 |
| | Chromatin-remodeling ATPase INO80 | Ino80 | 77.9 |
| | Actin-related protein 8 | Actr8 | 50.1 |
| | Cellular tumor antigen p53 | Tp53 | 47.2 |
| Sister chromatid cohesion (3) | | | |
| | Sister chromatid cohesion protein PDS5 homolog A | Pds5a | 192.7 |
| | Structural maintenance of chromosomes protein 1A | Smc1a | 176.9 |
| | Cohesin subunit SA-2 | Stag2 | 56.4 |

## B. TET1-*Platr10* interaction by CLIP

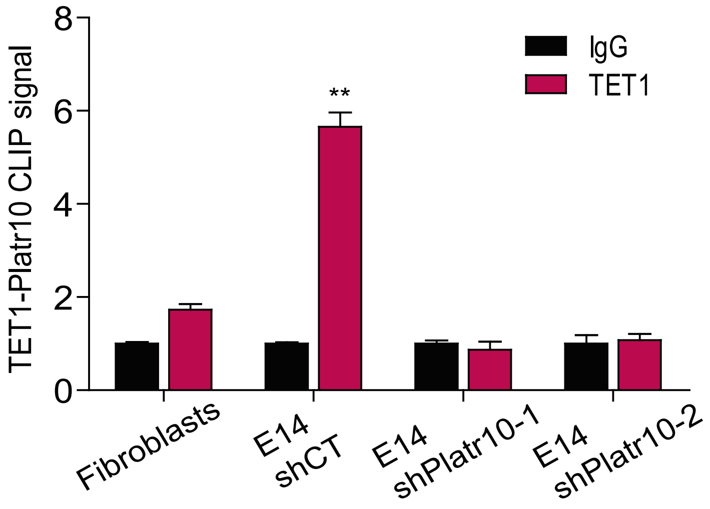


Figure S14. *Platr10*-TET1 interaction by CLIP and RNA pulldown-MS sequencing

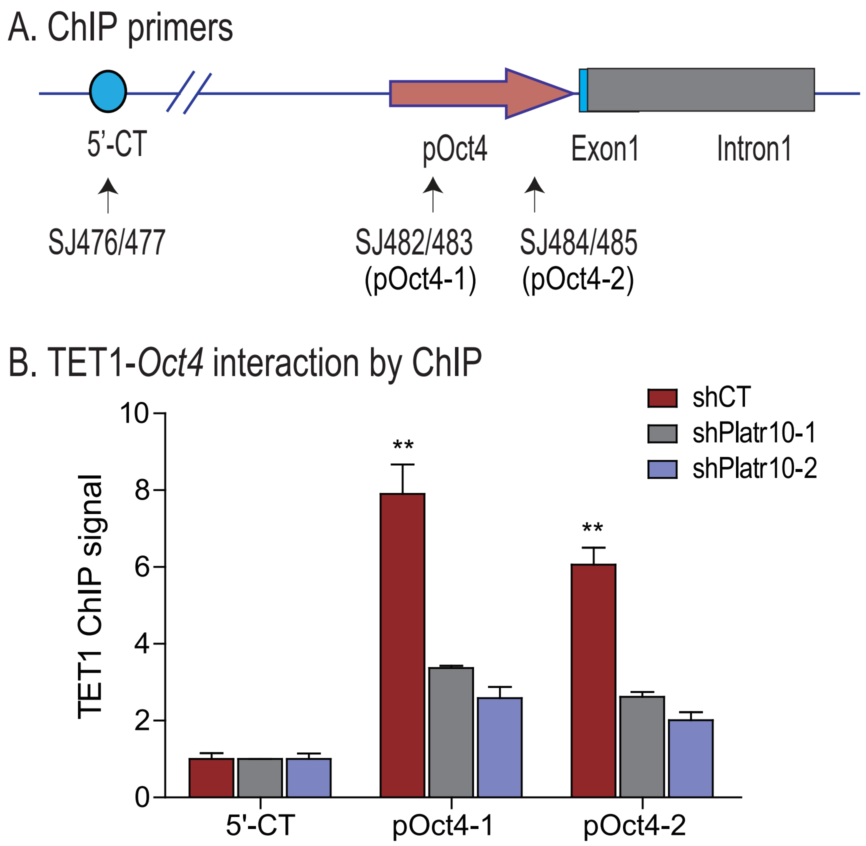


Figure S15. Interaction of TET1 with the *Oct4* promoter.

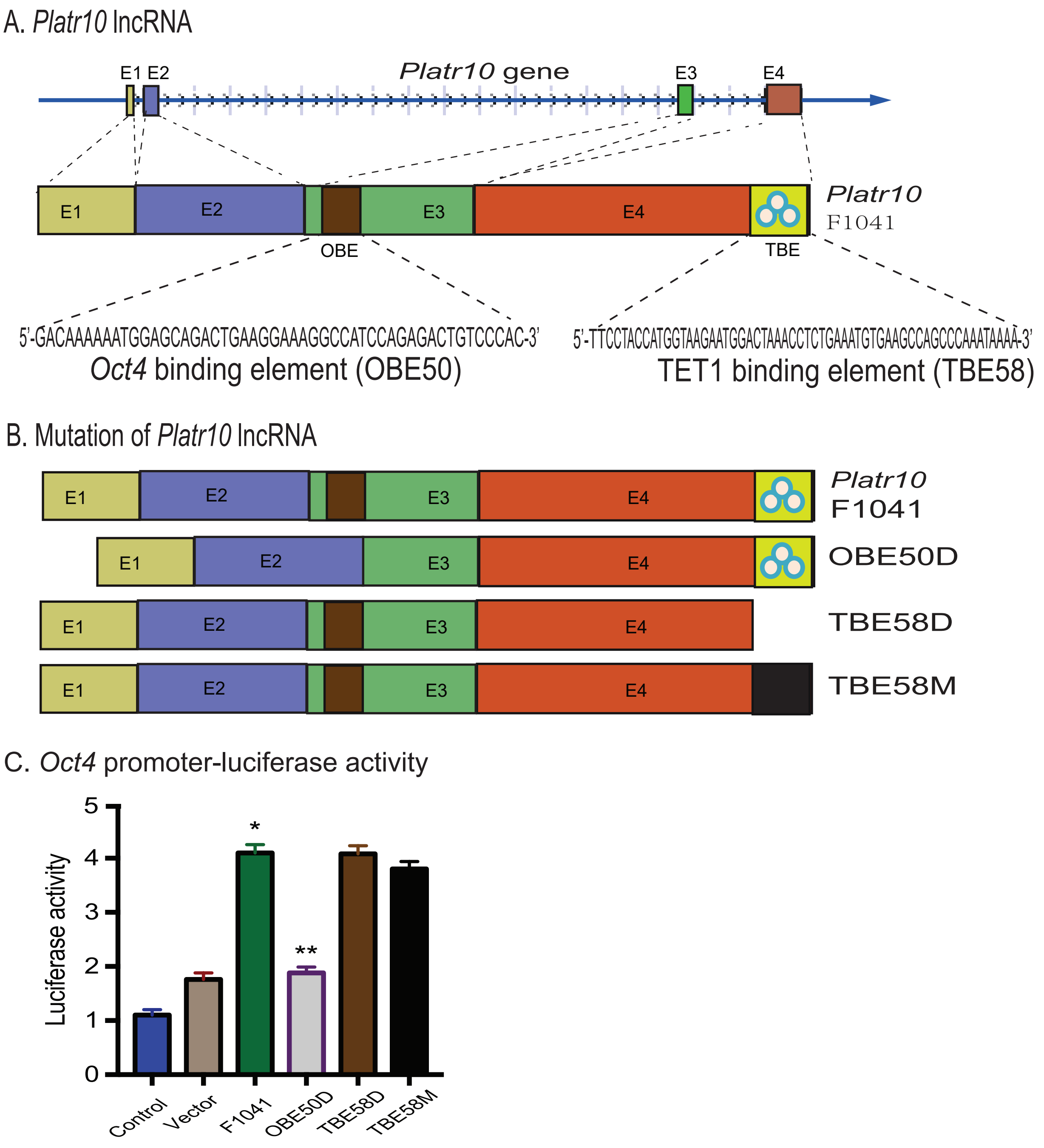


Figure S16. Mutation of *Platr10-OcT4* binding element and TET1 binding element

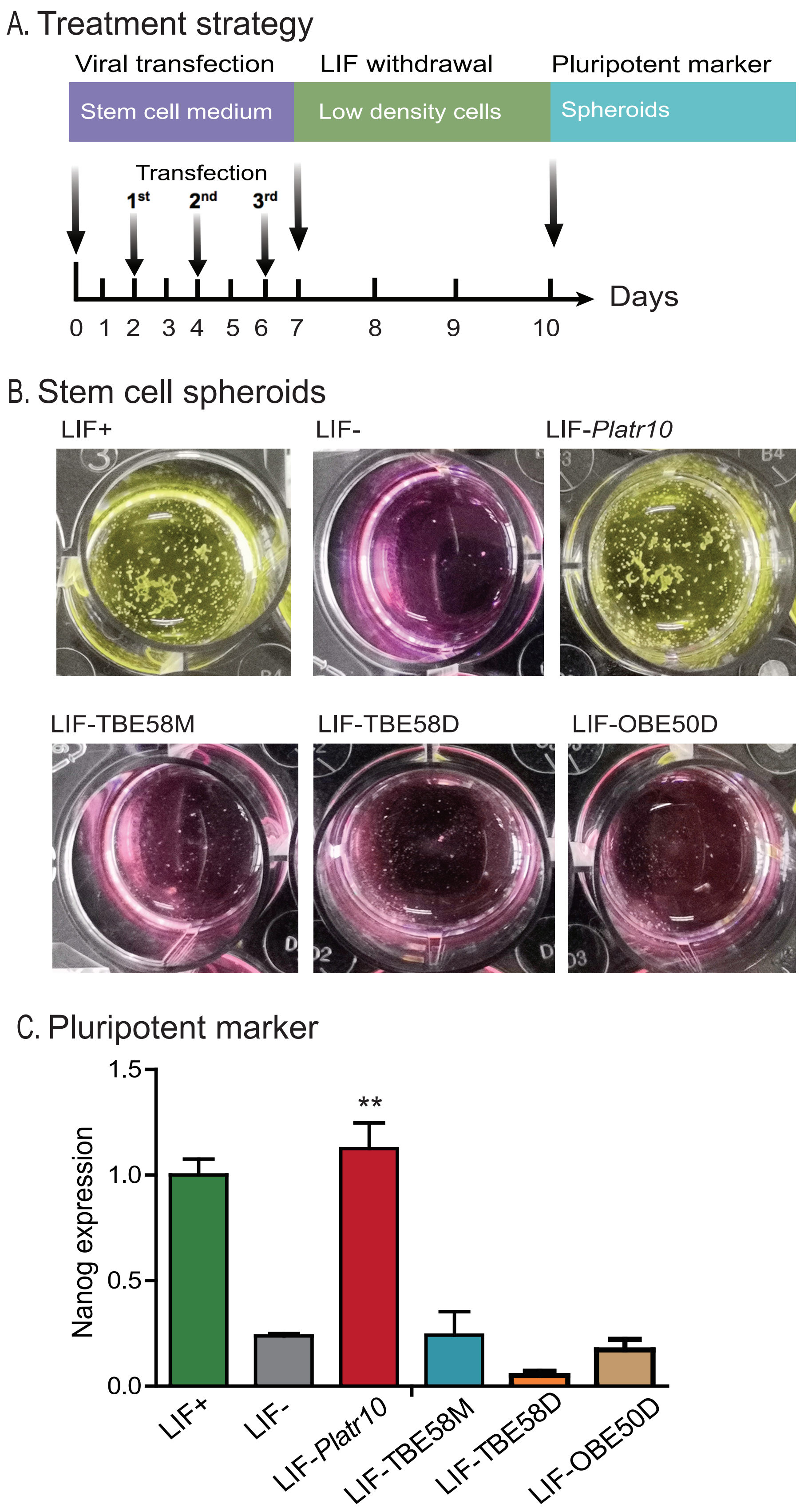


Figure S17. *Platr10* partially corrects the LIF withdrawal-induced defects

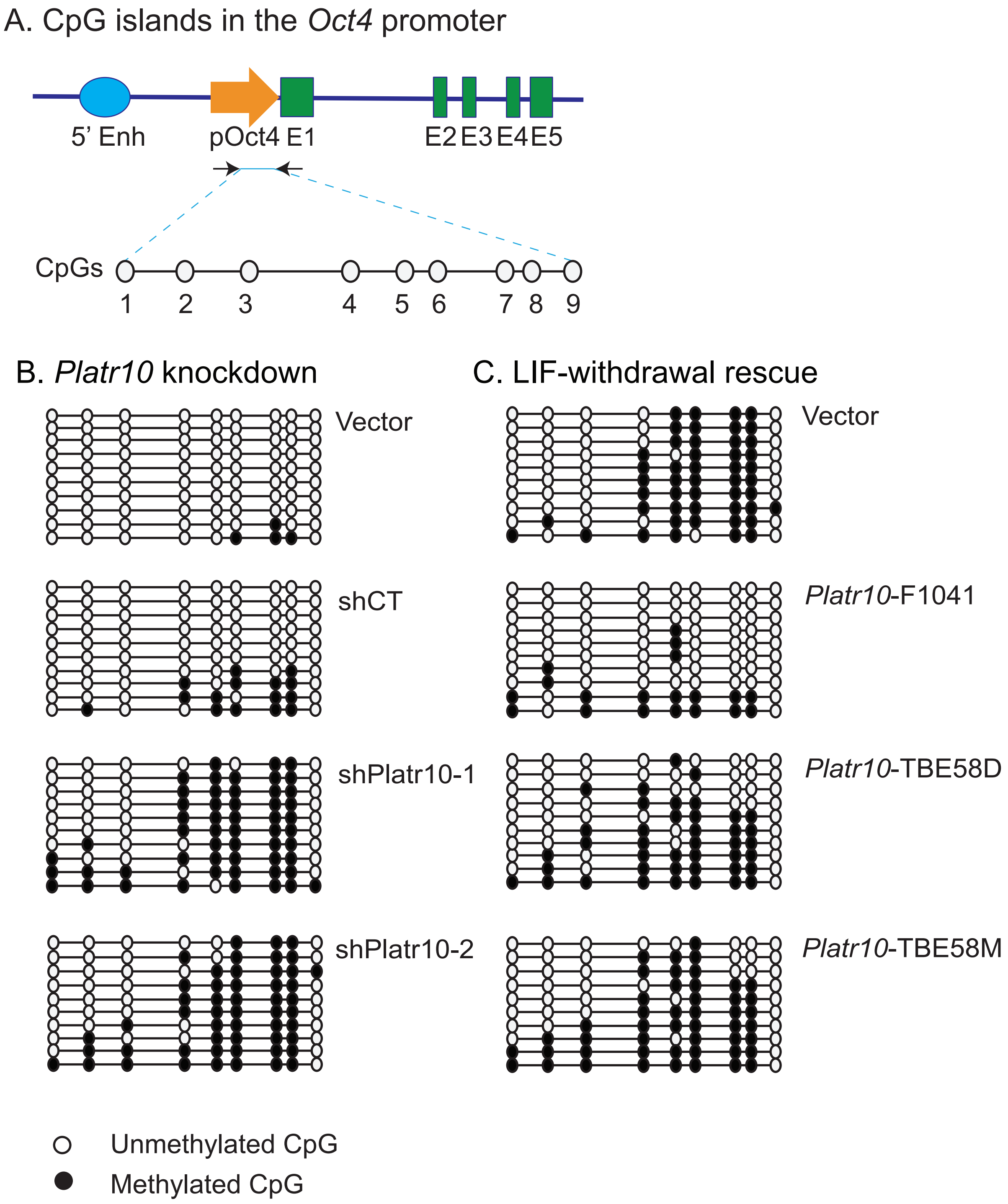


Figure S18. The role of *Platr10* and its mutants in *Oct4* DNA methylation
