# Supplementary material for: Chromatin lncRNA Platr10 controls stem cell pluripotency by coordinating an intrachromosomal regulatory network
Source: Genome Biol. 2021 Aug 19;22:233. doi: 10.1186/s13059-021-02444-6 (PMC8375132; doi:10.1186/s13059-021-02444-6)
Supplement: Supplementary file 2 — Additional file 2:. Review history [file 13059_2021_2444_MOESM2_ESM.docx]

**Review history**

**First round of review**

**Reviewer 1**

The authors describe a new molecular mechanism for the lcnRNA Platr10 in pluripotency.
They show that Platr10 binds to the regulatory elements of important pluripotency factors including Oct4 and Sox2, and that it impacts the local chromatin structure. The authors further demonstrate that Platr10 contains a TET binding element in addition to its Oct4 interaction element.

While the authors provide a compelling mechanism and apply a number of state-of-the-art high throughput sequencing technologies, a few open questions remain. While the title of the manuscript suggests that it focuses on intrachromosomal regulatory structures, this is only a small part of the paper that otherwise focuses more on the general role of Platr10 in pluripotency and its interaction with TET. Based on the data presented, it may be better to focus on the demethylation phenotype observed in the presence of Platr10, and include an experiment that shows a direct interaction of the Platr10-TET complex at the Oct4 promoter (and/or additional pluripotency genes). It might also be good to connect the TET/methylation part of the story better to the changes in chromatin looping, which currently seem parallel storylines rather than directly and functionally connected. The RNA-FISH data does not look convincing.

Detailed comments for the authors:
1. Line 184: The authors mention that Platr10 was identified based on RNA-seq, but do not give sufficient credit to the original publication. This lncRNA was identified in a pluripotency screen comparing ESCs and NPCs, and accordingly named Pluripotency associated transcript 10. The Bergmann et al paper is not simply a differential RNA-seq screen but performed elaborate computational analyses, including a weighted co-expression analysis, and identified 32 pluripotency-associated lncRNAs (Platrs) clustering tightly with Oct4 expression and other pluripotency factors. While the authors present a compelling mechanism for the lncRNA here, they are not the first group that identified an association with Oct4, differential expression in ESCs/iPSCs or role in pluripotency for Platr10. The authors also previously reported on Platr10 being bound to both Sox2 and Oct4 locus in their 2019 CRIST-seq paper.

2. Fig S9: Rather unusual display of cellular fractionation - unclear if this is a gel/blot, and why it was not quantified using qRT-PCR? In addition to nucleus vs cytoplasm, it would be good to show chromatin-bound vs nucleoplasm, especially given the strange RNA-FISH signal. The RNA-FISH signal seems to be very strong and diffuse across the nucleus, in fact it looks exactly like the DAPI image - is this a technical error, high background, or is Platr10 really 100% overlapping with DAPI signal and binds to all genes?

3. Fig 3A and S11 are repetitive.

4. How many target genes were identified in Platr10 RAT-seq in total? Related to my above comments on RNA-FISH, does PLatr10 bind a lot of different genes? The top hits do not seem to be pluripotency genes.

5. Fig S11: multiple typos, limited value of ontology figure as all terms are very generic. Fig S11C should not be a screen shot. Statistics for the motif enrichment are missing (fold-change compared to background, how many target sites have this motif vs how many sites exist in the entire genome, p-value). The significance of motif identification is not clear.

6. Fig 4: Differential chromatin loops could be displayed in a more compelling way. Sequencing tracks are not necessary in the main figure.

7. Regarding the proposed mechanism, it is essential to show that TET and Platr10 bind together at the e.g. Oct4 locus, and that TET binding and/or demethylation activity at the locus is Platr10 driven. A ChIP at the Oct4 locus in the presence or absence of Platr10 could answer this question.

8. How does Platr10 bind to chromatin? Triple-helix? R-loop? Protein mediated (TET)?

9. Similarly, how do methylation status and chromatin looping at the Oct4 locus impact each other? Is TET/methylation status involved in chromatin structure or is this driven by other factors such as CTCF/cohesin?

10. Fig S14 (and S15, S16) shows a TBE58D and TBE58M mutant, not 64 as described in the text

11. It is not clear form the methods how many replicates were carried out for RNA-seq (or other HTS assays), please clarify.

**Reviewer 2**

The study entitled "Chromatin lncRNA Platr10 controls stem cell pluripotency by coordinating intra-chromosomal regulatory network" combines several interesting techniques (e.g., CRIST-seq & RAT-seq) to determine which lncRNAs associate with OCT4 promoter. The authors demonstrate that CRIST-seq can be used to target the OCT4 promoter by subsequent FLAG-IP of CAS9, relative to a randomly localized CAS9. The authors then cross-link cells perform RT and then sequence lncRNAs enriched two fold over non-targeting (and IGG but that often is an incomplete control, but good the authors added for completeness). They then intersect this list with genes that are activated in directed reprogramming. This resulted in 27 lncRNAs that interact with OCT4 and SOX2 promoter and are induced in reprogramming. The authors focus on characterizing one candidate Platr10 that is a stem cell specifically expressed lncRNA. The authors test the loss of function of Platr10 by shRNA knockdown. Upon depletion of Platr10 stem cells show a differentiation defect relative to control shRNAs -- due to loss of Oct4, Sox2 and Nanog. Next gain of Platr10 function was tested by a lentiviral inducible system resulting in increased reprogramming. Using RAT-seq the authors find that Platr10 binds to OSKM promoters and using 3C. The authors used a supervised hypothesis approach to test if TET family proteins are involved in de-methylating the OCT4 promoter and perform RIP-PCR to find Platr10 bound to TET1. Moreover, they used a RIP strategy with extra shearing to determine fragment of Platr10 that bound. This was performed orthogonally using recombinant TET1 and RNA baits -- the authors identify a 57mer 3' nucleotide sequence that interacts with TET that is "the exact same" as the RIP assay. Impressively, the authors tested different mutations to determine if the TET1 binding element is required and found that it was not, but explain -- very reasonably -- that other elements are likely to be involved (Perhaps TET2).

Overall this study is a tour de force with numerous highly-technical experiments in each results section. The authors have applied numerous techniques to get to a detailed understanding of Platr10.  As such this study is certainly of interest to the broad readership of Genome Biology. However, I feel the following should be considered before publication.


1) ShRNA knockdown experiments. There are numerous issues with using shRNAs and even more arising recently (Basset et al. eLife 2014). Although the experiments were performed correctly the loss of function studies should be tested with orthogonal methods such as CRISPR-I, CRISPR deletion of promoter. Especially owing to nuclear expression of Platr10 it is hard to understand how shRNAs could be so effective in the nucleus. Moreover, the new findings that shRNA mediated knockdown results in epigenetic silencing of the locus. Thus this LOF experiment could be due to a cis DNA element in the Platr10 locus and not the RNA. Although the authors demonstrate that gain of function increases reprogramming they are not necessarily related processes as gain of function could affect other pathways (e.g., p53) -- that in turn increase reprogramming.

2) RAT-seq.

a) The authors describe OSKM factors from RAT-seq, however it is not reported how many other genes were found. How specific are these OSKM factors relative to the total sequenced pool?


b) In this section the technique is not described well enough to understand the target (DNA, RNA or protein). I ended up figuring it out after reading a few times, but should be explained in more detail. More minor, but the notion that the GO terms from RAT-seq are pluripotent is tautological as they as this was tested in a stem cell.

3) 3C is becoming an antiquated technique that is highly biased by the proximities tested here. Can the authors investigate mouse HiC data, that is at incredible resolution these days, to demonstrate these loops?

4) RIP-seq

a) RIP-seq of TET proteins is not clear if this initial data set is native or cross-linked? Regardless, it should be fully sequenced to know if Platr1 is highly enriched or one of many hundreds or thousands of RNAs. Are these RNAs mostly lncRNAs or equally distributed between lncRNAs and mRNAs? The global population is required to understand the relevance of Platr10. Especially considering the authors, reasonably so, picked TET1 to study -- yet if TET1 binds to hundreds or thousands of RNA it makes one wonder the specificity of Platr10?

b) The same argument applies for the shearing experiments to identify the TBE. How many other RNAs were found? Were there similar sequence motifs or different? This again brings into question the specificity if TET1 binds to hundreds or thousands of RNAs.

c) Clip and fRIP experiments have been performed in mESCs for SOX2 (Holmes et al. NatCom 2020). Was Platr10 also found in those studies? If not how can this be explained with more stringent CLiP conditions?

**Authors’ response to reviewers**

**Reviewer #1**:

*“They show that Platr10 binds to the regulatory elements of important pluripotency factors including Oct4 and Sox2, and that it impacts the local chromatin structure. The authors further demonstrate that Platr10 contains a TET binding element in addition to its Oct4 interaction element.*

*While the authors provide a compelling mechanism and apply a number of state-of-the-art high throughput sequencing technologies, a few open questions remain. While the title of the manuscript suggests that it focuses on intrachromosomal regulatory structures, this is only a small part of the paper that otherwise focuses more on the general role of Platr10 in pluripotency and its interaction with TET. Based on the data presented, it may be better to focus on the demethylation phenotype observed in the presence of Platr10, and include an experiment that shows a direct interaction of the Platr10-TET complex at the Oct4 promoter (and/or additional pluripotency genes). It might also be good to connect the TET/methylation part of the story better to the changes in chromatin looping, which currently seem parallel storylines rather than directly and functionally connected. The RNA- FISH data does not look convincing.”*

**Answer**: We thank the reviewer for the useful comment to improve our manuscript. In this revised version, we have performed a series of assays to address the epigenetic role and mechanisms of *Paltr10*, including the *Platr10*-TET1 interaction by CLIP and RNA pulldown-MS protein sequencing, the TET1-Oct4 interaction by ChIP, the *Platr10*-Oct4 interaction by ChIRP and RAT assays. Additionally, we have also repeated the RNA-FISH assay for *Platr10*. (See the following answers for detail).

1. *“Line 184: The authors mention that Platr10 was identified based on RNA-seq, but do not give sufficient credit to the original publication. This lncRNA was identified in a pluripotency screen comparing ESCs and NPCs, and accordingly named Pluripotency associated transcript 10. The Bergmann et al paper is not simply a differential RNA-seq screen but performed elaborate computational analyses, including a weighted co-expression analysis, and identified 32 pluripotency-associated lncRNAs (Platrs) clustering tightly with Oct4 expression and other pluripotency factors. While the authors present a compelling mechanism for the lncRNA here, they are not the first group that identified an association with Oct4, differential expression in ESCs/iPSCs or role in pluripotency for Platr10. The authors also previously reported on Platr10 being bound to both Sox2 and Oct4 locus in their 2019 CRIST-seq paper.”*

**Answer**: We agree with the reviewer. In the revised version, we have described the Bergmann paper (reference #16) in more detail in the Results section (p.8, para.3; p.9, para.1) and in the Introduction (p.5, para.3).

2. *“Fig S9: Rather unusual display of cellular fractionation - unclear if this is a gel/blot, and why it was not quantified using qRT-PCR? In addition to nucleus vs cytoplasm, it would be good to show chromatin-bound vs nucleoplasm, especially given the strange RNA-FISH signal. The RNA-FISH signal seems to be very strong and diffuse across the nucleus, in fact it looks exactly like the DAPI image - is this a technical error, high background, or is Platr10 really 100% overlapping with DAPI*

*signal and binds to all genes?”*

**Answer**: The data in Figure S9 are the RT-PCR gel image. As suggested, we have performed quantitative PCR for cellular distribution (Fig.S9B). The abundance of chromatin-bound and nucleoplasm *Platr10* has been quantitated in Figure S9C. We show that *Platr10* is present primarily in the chromatin-bound form.

We have also repeated the RNA-FISH assay for *Platr10*, and replaced the data with images that have a better resolution (Fig.S9D).

3. *“Fig 3A and S11 are repetitive.”*

**Answer**: We have deleted original Figure S10, which shows the schematic diagram of RAT-seq similar to Figure 3A.

4. *“How many target genes were identified in Platr10 RAT-seq in total? Related to my above comments on RNA-FISH, does PLatr10 bind a lot of different genes? The top hits do not seem to be pluripotency genes.”*

**Answer**: We apologize for not having given a clear description in the first submission. In RAT-seq, differential binding analysis was performed with the DiffBind package using parameters of fold- change difference >2 and p-value < 0.05, with false discovery rate (FDR) <0.1. Using these criteria, we identified 416 *Platr10* target genes. In the revised version, we have described this methodology in greater detail (p12, para.1).

It is true that *Platr10* also binds to many targets that are not known to be stemness genes. Currently, we are not sure whether those target genes are also involved in the establishment and maintenance of pluripotency. It is likely that *Platr10* may have multiple functions, in addition to its role of regulating pluripotent genes. We have discussed this issue in the revised text (p20, para.1).

In support to this concept, the new RNA pulldown-MS protein data also show that *Platr10* lncRNA binds to many factors in other pathways, like chromatin modification and remodeling, PI3K-Akt signaling, ribosome biosynthesis, and cell cycles (Fig.S14B).

5. *“Fig S11: multiple typos, limited value of ontology figure as all terms are very generic. Fig S11C should not be a screen shot. Statistics for the motif enrichment are missing (fold-change compared to background, how many target sites have this motif vs how many sites exist in the entire genome, p- value). The significance of motif identification is not clear.”*

**Answer**: Figure S11 has been deleted in the revised version due to its limited value.

6. *“Fig 4: Differential chromatin loops could be displayed in a more compelling way. Sequencing tracks are not necessary in the main figure.”*

**Answer**: We have revised Figure 4 as per Reviewer’s suggestion. The data of loop sequencing has been moved to supplementary materials (Fig.S13).

7. *“Regarding the proposed mechanism, it is essential to show that TET and Platr10 bind together at the e.g. Oct4 locus, and that TET binding and/or demethylation activity at the locus is Platr10 driven. A ChIP at the Oct4 locus in the presence or absence of Platr10 could answer this question.”*

**Answer**: We thank the reviewer for the useful suggestion. We have performed a ChIP assay to confirm the binding of TET1 to the *Oct4* locus. The new data have been added in Figure S15. In addition, we have added new data from ChIRP (Fig.S12B), RAT (Fig.S12A), CLIP (Fig.S14A), and RNA pulldown-MS protein mapping (Fig.S14B). All these new data support the *Platr10*-TET1-*Oct4* interaction.

8. *“How does Platr10 bind to chromatin? Triple-helix? R-loop? Protein mediated (TET)?”*

**Answer**: Currently, we know very little about how *Paltr10* recognizes its target genes, like *Oct4*. By searching the lncRNA-DNA interaction prediction website (LongTarget: <http://lncrna.smu.edu.cn/show/DNATriplex>), we could not find an interaction using the base matching mechanism between *Platr10* and the *Oct4* promoter.

To solve this issue, we have performed RNA pulldown-MS protein sequencing for *Platr10* lncRNA. The MS sequencing data show that in addition to TET1, *Platr10* also binds to a number of proteins that are involved in covalent chromatin modification, chromatin remodeling, and chromosome cohesion (Fig.S14B). Thus, it is possible that this lncRNA-chromatin interaction may require the participation of other chromatin factors, such as CTCF and Cohesin. In the revised manuscript, we have discussed these issues in the Discussion section (p.21, para.2).

9. *“Similarly, how do methylation status and chromatin looping at the Oct4 locus impact each other? Is TET/methylation status involved in chromatin structure or is this driven by other factors such as CTCF/cohesin?”*

**Answer**: It is not clear if the methylation status and chromatin looping at the *Oct4* locus impact each other. However, the data form RNA pulldown-MS sequencing suggest that *Platr10* binds multiple factors involved in covalent chromatin modification and chromatin remodeling (Fig.S14B), including TET1, TET2, SMC1a, CTCF, Cohesin subunit SA-2 (Stag2), EZH2, histone chaperone ASF1A, histone acetyltransferases (KAT7, KAT8, KAT6b), lysine-specific demethylase 3B, Sin3 histone deacetylase corepressor complex component SDS3, and although it seems that *Platr10* does not bind to MED1. Thus, it is likely that by binding to these chromatin factors, *Platr10* lncRNA may coordinate methylation status and chromatin looping at the *Oct4* locus. Further studies are needed to address these critical issues. We have discussed this issue in the revised text (p.19, para.1; p21, para.2).

10. *“Fig S14 (and S15, S16) shows a TBE58D and TBE58M mutant, not 64 as described in the text.”*

**Answer**: A 64 bp fragment was initially identified by RIP sequencing. Due to the presence of some shorter *Platr10* RNA transcripts from 3’-RACE, a final 58 bp consensus TBE element was used for further studies (p.17, para.1). We have corrected the typos in these figures and the text.

11. *It is not clear form the methods how many replicates were carried out for RNA-seq (or other HTS assays), please clarify.*

**Answer**: We apologize for not having described clearly it in our first submission. In the revised version, we have added them in more detail (p.25, para.2; p.29, para.1; Figure 2 legend, p.45, para.3).

# Reviewer #2:

*“The study entitled "Chromatin lncRNA Platr10 controls stem cell pluripotency by coordinating intra-chromosomal regulatory network" combines several interesting techniques (e.g., CRIST-seq & RAT-seq) to determine which lncRNAs associate with OCT4 promoter. The authors demonstrate that CRIST-seq can be used to target the OCT4 promoter by subsequent FLAG-IP of CAS9, relative to a randomly localized CAS9. The authors then cross-link cells perform RT and then sequence lncRNAs enriched two fold over non-targeting (and IGG but that often is an incomplete control, but good the authors added for completeness). They then intersect this list with genes that are activated in directed reprogramming. This resulted in 27 lncRNAs that interact with OCT4 and SOX2 promoter and are induced in reprogramming. The authors focus on characterizing one candidate Platr10 that is a stem cell specifically expressed lncRNA. The authors test the loss of function of Platr10 by shRNA knockdown. Upon depletion of Platr10 stem cells show a differentiation defect relative to control shRNAs -- due to loss of Oct4, Sox2 and Nanog. Next gain of Platr10 function was tested by a lentiviral inducible system resulting in increased reprogramming. Using RAT-seq the authors find that Platr10 binds to OSKM promoters and using 3C. The authors used a supervised hypothesis approach to test if TET family proteins are involved in de-methylating the OCT4 promoter and perform RIP-PCR to find Platr10 bound to TET1. Moreover, they used a RIP strategy with extra shearing to determine fragment of Platr10 that bound. This was performed orthogonally using recombinant TET1 and RNA baits -- the authors identify a 57mer 3' nucleotide sequence that interacts with TET that is "the exact same" as the RIP assay. Impressively, the authors tested different mutations to determine if the TET1 binding element is required and found that it was not, but explain -- very reasonably -- that other elements are likely to be involved (Perhaps TET2).”*

*“Overall this study is a tour de force with numerous highly-technical experiments in each results section. The authors have applied numerous techniques to get to a detailed understanding of Platr10. As such this study is certainly of interest to the broad readership of Genome Biology.*

*However, I feel the following should be considered before publication.”*

**Answer**: We thank the reviewer for the encouraging comments on our manuscript. We have performed a series of new assays to address the concern.

1). *“ShRNA knockdown experiments. There are numerous issues with using shRNAs and even more arising recently (Basset et al. eLife 2014). Although the experiments were performed correctly the loss of function studies should be tested with orthogonal methods such as CRISPR-I, CRISPR deletion of promoter. Especially owing to nuclear expression of Platr10 it is hard to understand how shRNAs could be so effective in the nucleus. Moreover, the new findings that shRNA mediated knockdown results in epigenetic silencing of the locus. Thus this LOF experiment could be due to a cis DNA element in the Platr10 locus and not the RNA. Although the authors demonstrate that gain of function increases reprogramming they are not necessarily related processes as gain of function could affect other pathways (e.g., p53) -- that in turn increase reprogramming.”*

**Answer**: As suggested, we have performed the loss-of-function assay using CRISPR Cas9 deletion. We used two vectors carrying the dual SpCas9-NmCas9 cassette and the gRNAs targeting the *Platr10* promoter and 3’-downstream region. Using a donor vector carrying two *Platr10* arms for homologous recombination, copGFP/Puro for positive selection, and TK for negative selection, we successfully deleted the *Platr10* promoter and coding region in E14 cells. The genomic deletion of *Platr10* was confirmed by DNA sequencing and pluripotency was examined by stemness marker HIC. These new data have been added in Figure S8 (p.11, para.2)

We agree with the reviewer that other pathways, like *p53*, may be involved in the role of *Platr10* in enhancing reprogramming. Our RNA pulldown-MS sequencing data suggest that in addition to factors involved in covalent chromatin modification and chromatin remodeling, *Platr10* also directly binds to P53 (Fig.S14B). Thus, it is possible that the nuclear *Platr10* may enhance reprogramming, at least partially, by regulating the P53 pathway. We have discussed this issue in the revised text (p21, para.2).

2). *“RAT-seq.*
a) *The authors describe OSKM factors from RAT-seq, however it is not reported how many other genes were found. How specific are these OSKM factors relative to the total sequenced pool?”*

**Answer**: We apologize for the confusion. After adjusting over the random control, we identified 416 target genes for *Platr10* (p12, para.1).

We believe that the OSKM binding is *Paltr10*-specific. First, the RAT-seq assay includes the random oligo and control lncRNA groups, which were run side-by-side with the *Platr10*-specific oligos. We did not detect the binding signal of OSKM in the random control group, or in either of the two control lncRNA groups (*Palr35* and *Palr34*). Second, *Platr10* did not bind to other stemness genes, like *Nanog*. Further studies are needed to address the mechanisms by which *Platr10* regulates its other target genes, including SKM.

b) *“In this section the technique is not described well enough to understand the target (DNA, RNA or protein). I ended up figuring it out after reading a few times, but should be explained in more detail. More minor, but the notion that the GO terms from RAT-seq are pluripotent is tautological as they as this was tested in a stem cell.”*

**Answer**: We have clarified the confusion by describing the Method (p29, para.1) and Figure 3 legend (p.47, para.1) in more detail. In this communication, RAT-seq was used to identify the genome-wide binding targets of *Platr10*, although the approach may also be used to identify the lncRNA-interacting proteins when in combination with MS.

We have deleted Figure S11 that contains the GO pathway due to the limited value as addressed by Reviewer #1.

3) *“3C is becoming an antiquated technique that is highly biased by the proximities tested here. Can the authors investigate mouse HiC data, that is at incredible resolution these days, to demonstrate these loops?”*

**Answer**: We thank the reviewer for the useful comment. Several groups have performed the HiC assay for mouse and human pluripotent stem cells. However, as seen in the following figure, the resolution in those Hi-C data is too low to delineate the intrachromosomal interaction at the *Oct4* locus. Our collaborators are working on the Micro-C data in mESCs, hoping that it will resolve this issue. In the revised text, we have discussed this issue and cited those Hi-C data sources (p.20, para.2; p.21, para.1).

|  | **Figure. Hi-C at the *Oct4***  **locus.**   A. Hi-C at the mouse Oct4 locus.   Data source: Bonev B et al. Multiscale 3D Genome Rewiring during Mouse Neural Development. Cell 2017:171:557-572.e24   B. Hi-C at the mouse Oct4 locus.   Data source: Dixon JR et al. Chromatin architecture reorganization during stem cell  differentiation. Nature 20  15 Feb  19;518(7539):331-6. |
| --- | --- |

4) *“RIP-seq*
   a) *RIP-seq of TET proteins is not clear if this initial data set is native or cross-linked? Regardless, it should be fully sequenced to know if Platr1 is highly enriched or one of many hundreds or thousands of RNAs. Are these RNAs mostly lncRNAs or equally distributed between lncRNAs and mRNAs? The global population is required to understand the relevance of Platr10. Especially considering the authors, reasonably so, picked TET1 to study -- yet if TET1 binds to hundreds or thousands of RNA it makes one wonder the specificity of Platr10?”*

**Answer**: We apologize for the confusion. The data were derived from the formaldehyde-crosslinked E14 cells. We have clarified the confusion in the revised text (p.34, para.2; p.48, para.2). The CLIP assay was not cross-linked (Fig.S14A).

The TET family methyldioxygenases (TET1, TET2, and TET3) are known to catalyze hydroxylation of 5- methylcytosine to generate 5-hydroxymethylcytosine in DNA. Quite recently, Dr. Francois Fuks’s group revealed that both TET1 and TET2 were critical for hydroxymethylation in RNA too (Lan J, Nature Commun 2020;11:4956). The TET family had an RNA-binding domain at the C- terminal. Using RIP-seq, they showed that TET1 bound to a total of 7798 RNA targets and TET2 bound to 6659 RNA transcripts. Most of them are coding mRNAs, and some are lncRNAs. By looking at their RIP-seq data, we found that *Platr10* (2410007B07Rik) was ranked 936 in the RNA list.

We have also confirmed this interaction with a CLIP assay (Fig.S14B). In addition, we have performed an RNA pulldown-MS sequencing approach to map the protein targets for *Platr10* (Fig.S14B). Nonetheless, it seems that TET1 catalyzes hydroxylation in both DNA and RNA, and *Platr10* is just one of its targets. We have discussed this issue in the revised test (p.22, para.1).

b) *“The same argument applies for the shearing experiments to identify the TBE. How many other RNAs were found? Were there similar sequence motifs or different? This again brings into question the specificity if TET1 binds to hundreds or thousands of RNAs.”*

**Answer**: We appreciate the reviewer’s comment. Further studies are needed to address if TET1- interacting RNA molecules have a consensus motif as in TBE. We have discussed this issue in the revised test (p.22, para.1).

c) *“Clip and fRIP experiments have been performed in mESCs for SOX2 (Holmes et al. Nat Com 2020). Was Platr10 also found in those studies? If not how can this be explained with more stringent CLiP conditions?”*

**Answer**: In that study, Holmes et al used both CLIP and formaldehyde cross-linked immunoprecipitation (fRIP) approaches to map SOX2-binding RNA molecules in mECSs. They reported that fRIP-seq enriched 1010 genes, while the CLIP-seq approach enriched 54 genes, with 41 genes overlapping in both approaches. Clearly, the CLIP approach was more stringent than fRIP in identifying SOX2-binding RNAs. The fRIP-seq approach, on the other hand, may include those RNA molecules that indirectly bind to SOX2.

However, it is surprising that they did not list those enriched genes in the supplementary materials and data sources. By analyzing their geo data, it seems that *Platr10* was in both fRIP-seq and CLIP- seq databases, suggesting an interaction of SOX2 with *Platr10* lncRNA. We have discussed this issue and cited the paper in the revised text (p.22, para.2).

We have performed the CLIP assay for *Platr10* (Fig.S14A). In addition, we have also used an RNA pulldown-MS sequencing approach to profile the protein molecules that interact with *Platr10*. We also detected the binding of *Platr10* lncRNA with SOX2, but not with other stemness marker proteins, like OCT4, NANOG, and KLF4 (Fig.S14B). Thus, it would be interesting to examine if in addition to the role of coordinating intrachromosomal looping at the *Oct4* locus, *Platr10* lncRNA may regulate pluripotency through modulating the activity of these pluripotent markers at the post- transcriptional level.

**Second round of review**

**Reviewer 1**

The authors have addressed most of my comments in a satisfactory manner. In particular, the TET1-Oct4-Platr10 connection has been solidified.

A couple of minor things to consider prior to publication:
- There are a lot of supplementary Figures, and only little data in some of the main figures, eg the revised Figure 4. The authors may wish to move some of the more important new data into the main figures ad strike a better balance between main figures and supplement.
- The Platr10 pull-down mass spec data (S14) shows log P values but not fold changes, which would be also useful. It seems that Dnmt1 binds as significantly as TET1 - this seems interesting in a methylation context. The authors may want to discuss this.

**Reviewer 2**

The authors have more than addressed my questions. I really appreciate them going above and beyond for each point made. Especially re-analyzing the data from Holmes et al. since the list of genes was not available. This and many other examples of numerous new experiments and analyses makes this suitable for publication in Genome Biology.

**Authors’ response to reviewers**

**Reviewer #1**: *“The authors have addressed most of my comments in a satisfactory manner. In particular, the TET1-Oct4-Platr10 connection has been solidified.

1. A couple of minor things to consider prior to publication:*
*- There are a lot of supplementary Figures, and only little data in some of the main figures, eg the revised Figure 4. The authors may wish to move some of the more important new data into the main figures ad strike a better balance between main figures and supplement.”*

**Answer**: We thank the reviewer for the constructive suggestion. As suggested, we have integrated some *Platr10*-binding protein MS mapping data in Figure 4C (p.48, para.5).

*2. –“The Platr10 pull-down mass spec data (S14) shows log P values but not fold changes, which would be also useful. It seems that Dnmt1 binds as significantly as TET1 - this seems interesting in a methylation context. The authors may want to discuss this.”*

**Answer**: We agree with the reviewer for the comment. It is interesting to see that in addition to the TET1 and TET2 DNA demethylase family, Platr10 also binds to DNA methyltransferase DNMT1. This finding suggests that by binding to DNMT1, *Platr10* may also regulate stem cell fate by actively coordinating DNA demethylation in many other target genes as identified by RAT-seq. In the revised version, we have discussed this finding as suggested (p.19, para.1; p.20, para.1).

**Reviewer #2**: *“The authors have more than addressed my questions. I really appreciate them going above and beyond for each point made. Especially re-analyzing the data from Holmes et al. since the list of genes was not available. This and many other examples of numerous new experiments and analyses makes this suitable for publication in Genome Biology.”*

**Answer**: We thank the reviewer for approving our manuscript.
